# PRMT-7/PRMT7 activates HLH-30/TFEB to guard plasma membrane integrity compromised by bacterial pore-forming toxins

**DOI:** 10.1080/15548627.2024.2306655

**Published:** 2024-01-23

**Authors:** Hui-Chen Hsieh, I-Hsiang Huang, Shao-Wen Chang, Po-Lin Chen, Yu-Cheng Su, Shuying Wang, Wei-Jiun Tsai, Ping-Hung Chen, Raffi V. Aroian, Chang-Shi Chen

**Affiliations:** a Department of Biochemistry and Molecular Biology, College of Medicine, National Cheng Kung University, Tainan, Taiwan; b Institute of Basic Medical Sciences, College of Medicine, National Cheng Kung University, Tainan, Taiwan; c Department of Medicine, College of Medicine, National Cheng Kung University, Tainan, Taiwan; d Department of Microbiology and Immunology, College of Medicine, National Cheng Kung University, Tainan, Taiwan; e Institute of Biochemistry and Molecular Biology, College of Medicine, National Taiwan University, Taipei, Taiwan; f Program in Molecular Medicine, University of Massachusetts Chan Medical School, Worcester, MA, USA

**Keywords:** Intrinsic cellular defense (INCED), plasma membrane integrity (PMI), pore-forming toxin (PFT), protein arginine methyltransferase-7 (PRMT7/PRMT-7), transcription factor EB (TFEB/HLH-30)

## Abstract

Bacterial pore-forming toxins (PFTs) that disrupt host plasma membrane integrity (PMI) significantly contribute to the virulence of various pathogens. However, how host cells protect PMI in response to PFT perforation *in vivo* remains obscure. Previously, we demonstrated that the HLH-30/TFEB-dependent intrinsic cellular defense (INCED) is elicited by PFT to maintain PMI in *Caenorhabditis elegans* intestinal epithelium. Yet, the molecular mechanism for the full activation of HLH-30/TFEB by PFT remains elusive. Here, we reveal that PRMT-7 (protein arginine methyltransferase-7) is indispensable to the nuclear transactivation of HLH-30 elicited by PFTs. We demonstrate that PRMT-7 participates in the methylation of HLH-30 on its RAG complex binding domain to facilitate its nuclear localization and activation. Moreover, we showed that PRMT7 is evolutionarily conserved to regulate TFEB cellular localization and repair plasma damage caused by PFTs in human intestinal cells. Together, our observations not only unveil a novel PRMT-7/PRMT7-dependent post-translational regulation of HLH-30/TFEB but also shed insight on the evolutionarily conserved mechanism of the INCED against PFT in metazoans.

## Introduction

Plasma membranes, which confine cells from their surroundings, are constantly exposed to a variety of menaces, including bacterial pore-forming toxins (PFTs), that imperil their vital barrier function with fatal repercussions [1]. Many bacterial pathogens, both gram-positive and negative, secrete PFTs that compromise the plasma membrane integrity (PMI) of host cells and are crucial for their infection and pathogenesis. Thus, from the perspective of host cells, sealing pores in the plasma membrane to maintain PMI is pivotal for cellular homeostasis in response to these noxious assaults in this predator-prey interaction [2]. Several cellular pore repair mechanisms against PFT perforation have been suggested, mostly by *in vitro* studies [2,3]. However, mechanistic investigation of the intrinsic cellular defense (INCED) of hosts at the organismal level and *in vivo* to this important class of bacterial virulence factors remains an under-researched area.

*Bacillus thuringiensis* crystal (Cry) toxins, including Cry5B and Cry21A applied in this study, are PFTs that can perforate the plasma membrane of intestinal cells of a variety of nematodes, including *Caenorhabditis elegans* [4,5]. This Cry toxin-*C. elegans* interaction system has rendered it feasible to investigate the mechanisms of action of bacterial PFT in a genetically trackable animal model *in vivo* and has led to the discovery of an array of significant INCED signaling pathways and cellular systems [5–7]. It is noteworthy that many of the mechanisms that defend *C. elegans* against Cry PFTs exhibit evolutionarily conserved responses in human cells under PFT attack. We and others have demonstrated that the host transcription factor EB (TFEB, orthologous to HLH-30 in *C. elegans*) is elicited by bacterial pathogens and their virulent components, including PFTs, to induce innate immunity for host protection [5,8–12]. Specifically, we demonstrated that nuclear translocation of HLH-30/TFEB from the cytoplasm is robustly elicited by Cry5B-PFT and the subsequent HLH-30/TFEB-dependent intrinsic cellular defense systems, including macroautophagy/autophagy, are significantly upregulated to maintain PMI in *Caenorhabditis elegans* intestinal cells [5]. However, the molecular mechanism for the full nuclear translocation of HLH-30/TFEB induced by PFT remains largely unknown.

HLH-30/TFEB, a member of the microphthalmia (MiT) family of basic helix-loop-helix leucine zipper (bHLH-Zip) transcription factors, is a master regulator for lysosome biogenesis and autophagy activation. The activity of TFEB can be regulated rigorously through transcription, post-transcriptional regulation, post-translational regulation, protein-protein interaction, and spatial organization in response to various cellular stimuli [13–17]. Moreover, subcellular localization of TFEB is primarily regulated by the MTOR (mechanistic target of rapamycin kinase) complex 1 (MTORC1)-mediated phosphorylation through the conserved RRAG GTPases-MTORC1-YWHA/14-3-3 sequester system [18,19]. Except for the phosphorylation by MTORC1, TFEB can also be regulated by the other kinase-dependent phosphorylations, and the other post-translational modifications (PTMs), including ubiquitination [20], acetylation [21], and glutathionylation [22]. Intriguingly, it has been demonstrated that protein nuclear localization can also be regulated by post-translational arginine methylation [23,24], and an arginine residue (arginine 8) located in the RRAGA binding domain of TFEB has been reported to be methylated in human intestinal cells [25]. These reports together prompted us to investigate whether the function of TFEB could also be regulated by the specific PTM, arginine methylation.

Protein arginine methyltransferases (PRMTs), encoded by the *prmt* genes, are a family of enzymes responsible for post-translational methylation on arginine residues. Methylations of arginine residues by PRMTs have been reported to participate in the regulation of several fundamental cellular processes, including transcription, RNA processing, and signal transduction [26]. Thus, dysregulations of PRMTs are reported in the development and progression of many human diseases [26]. However, whether a PRMT may mediate the PTM of HLH-30/TFEB remains unreported. Herein, we unveil that PRMT-7 is indispensable for the arginine methylation in the RRAGA binding domain of HLH-30 induced by bacterial PFT. Moreover, this specific PTM is required for the full nuclear localization of HLH-30/TFEB elicited by PFT and the subsequent activation of INCED systems to maintain PMI compromised by PFT. Finally, aberrant regulations in PRMT7 and TFEB have been linked to many human diseases [17,27]. Thus, understanding more about the PRMT7-TFEB signal transduction network may shed light on the molecular basis of these diseases and provide novel therapeutic targets for drug development.

## Results

### PRMT-7 is indispensable for the full HLH-30 nuclear localization elicited by Cry5B-PFT in C. elegans

**Figure 1.**
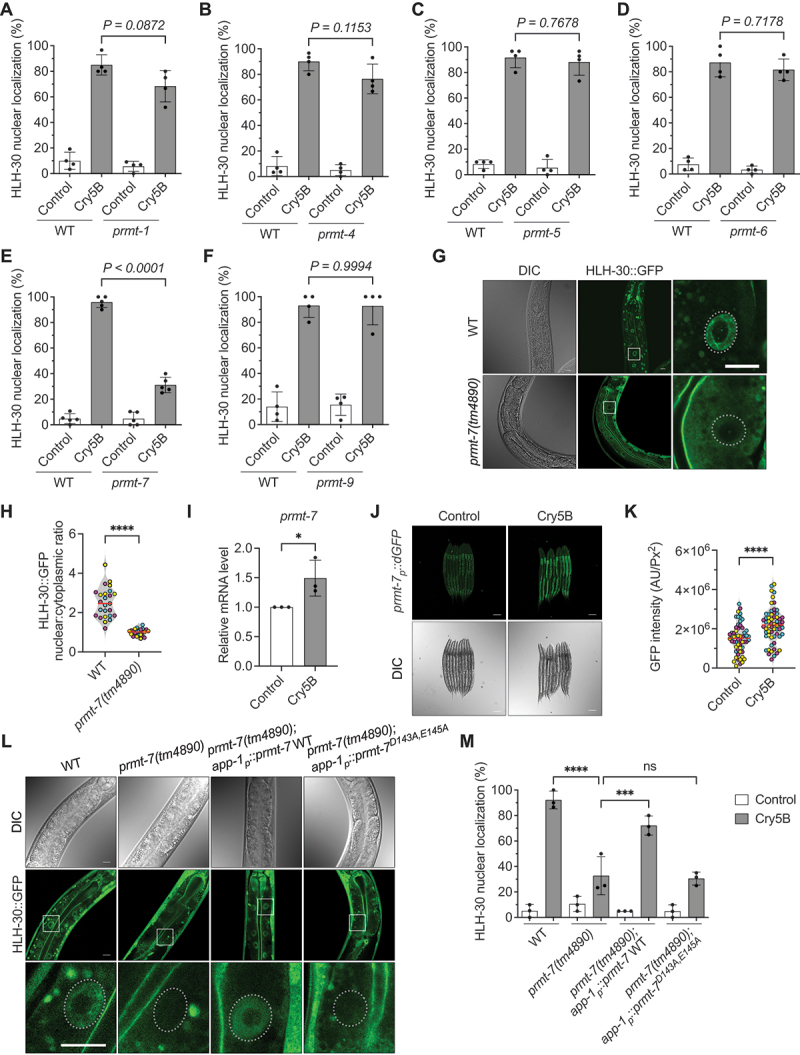
PRMT-7 is required for the full HLH-30 transnucleation induced by Cry5B-PFT in *C. elegans*. (A-F) the percentage of animals with HLH-30:GFP nuclear localization in the YQ348 *prmt-1(tm3613)* (*n* = 120, *N* = 4), YQ339 *prmt-4(tm4959)* (*n* = 120, *N* = 4), YQ352 *prmt-5(tm6220)* (*n* = 120, *N* = 4), YQ351 *prmt-6(tm5240)* (*n* = 120, *N* = 4), YQ340 *prmt-7(tm4890)* (*n* = 150, *N* = 5) and YQ353 *prmt-9(tm62933)* (*n* = 120, *N* = 4) mutants compared to the OP433 wild-type (WT) exposed to Cry5B or control for 1 h. (G) the representative DIC and confocal images of HLH-30:GFP in the wild-type (WT) and *prmt-7(tm4890)* mutant fed with Cry5B for 1 h. The enlarged images (right panels) are from the frame on the middle panel images of HLH-30:GFP. The dotted circles represent nuclear areas in the enlarged images. Scale bars: 10 μm. (H) the quantification of the nuclear:cytoplasmic ratio of the HLH-30:GFP intensity in WT (*n* = 25) and *prmt-7(tm4890)* animals (*n* = 23) fed with Cry5B for 1 h. (I) the qRT-PCR analysis of *prmt-7* mRNA in N2 wild-type animals fed with control (*n* = 9, *N* = 3) or Cry5B (*n* = 9, *N* = 3) for 3 h. (J) the representative image of YQ432 *prmt-7*_*p*_*::dGFP* (fast degradable GFP) transgenic animals exposed to Cry5B or control for 3 h. Scale bars: 100 μm. (K) the quantification of GFP intensity in YQ432 *prmt-7*_*p*_*::dGFP* animals exposed to control (*n* = 58) or Cry5B (*n* = 60) for 3 h. (L) the representative image of HLH-30:GFP in WT, YQ340 *prmt-7(tm4890)*, YQ483 *prmt-7(tm4890);app-1*_*p*_*::prmt-7* WT, and YQ482 *prmt-7(tm4890);app-1*_*p*_*::prmt-7*^*D143AE145A*^ animals fed with Cry5B for 1 h. Enlarged images (lower panels) are from the frame on the middle panel images of HLH-30:GFP. The dotted circles indicate nuclear areas in the enlarged images. Scale bars: 10 μm. (M) the percentage of worms with HLH-30 nuclear localization in WT (*n* = 90, *N* = 3), YQ340 *prmt-7(tm4890)* (*n* = 90, *N* = 3), YQ483 *prmt-7(tm4890);app-1*_*p*_*::prmt-7* WT (*n* = 90, *N* = 3), and YQ482 *prmt-7(tm4890);app-1*_*p*_*::prmt-7*^*D143A,E145A*^ (*n* = 90, *N* = 3) animals fed with Cry5B for 1 h. Data information: fig. 1A-1F and 1 M statistics based on: ***p < 0.001 and ****p < 0.0001 by two-way ANOVA. Fig. 1 H, 1I, and 1K statistics based on: *p < 0.05 and ****p < 0.0001 by unpaired *t* test (two-tailed). ns represents non-significance. Means are shown in red lines. Each data set of an independent biological repeat was represented by a different color. See also fig. S1. Source data are available online for this figure.

There are total of six *prmt* genes, including *prmt-1, prmt-4, prmt-5, prmt-6, prmt-7*, and *prmt-9*, currently identified in *C. elegans* (WormBase: WS288) [28,29]. To investigate the potential role of PRMTs in HLH-30 activation, we generated all six different *prmt C. elegans* mutants expressing HLH-30:GFP. These transgenic *C. elegans* were fed with the pore-forming toxin (PFT), Cry5B, for 1 h to induce the robust nuclear localization of HLH-30 in their intestinal cells (Figures 1A–F, and S1A-1 G). Our results showed that Cry5B-PFT significantly induced HLH-30:GFP nuclear localization in the intestinal cells of wild-type (WT), *prmt-1(tm3613)*, *prmt-4(tm4959)*, *prmt-5(tm6220)*, and *prmt-6(tm5240)* worms, however the percentage of worms with nuclear HLH-30:GFP induced by Cry5B was significantly diminished in *prmt-7(tm4890)* worms, with the presumable *prmt-7* loss-of-function mutation (WormBase: WS288) (Figure 1E and S1F). Also, compared to the wild-type, the intensity of nuclear HLH-30:GFP signals was significantly reduced in the *prmt-7(tm4890)* mutant (Figure 1G; the nucleus-to-cytoplasmic (N:C) ratio of HLH-30:GFP signals was quantified in 1 H). Interestingly, the expression levels of intestinal HLH-30:GFP protein and endogenous *hlh-30* mRNA were not affected in the *prmt-7(tm4890)* mutant animals compared to the WT (Fig. S1H-1J). Together these findings suggested that *prmt-7* plays an important role in controlling the cellular redistribution of HLH-30 caused by Cry5B-PFT, probably at the post-translational level.

Next, we examined whether Cry5B-PFT could activate *prmt-7* expression in *C. elegans*. We first analyzed the endogenous mRNA level of *prmt-7* by quantitative real-time reverse transcription polymerase chain reaction (qRT-PCR) analysis. Our results showed that the *prmt-7* mRNA level was significantly upregulated in N2 wild-type *C. elegans* animals in response to Cry5B intoxication (Figure 1I). We next generated a transgenic *C. elegans* strain expressing fast degradable GFP (dGFP) driven by the *prmt-7* promoter to monitor the expression tissue of *prmt-7* induced by PFT. The transcriptional reporter analysis indicated that Cry5B significantly increased dGFP signals, mostly in the intestinal cells (the primary Cry5B-PFT target cells [30]), in the *prmt-7*_*p*_*::dGFP* transgenic worms after Cry5B treatment (Figure 1J,K). Together, our data suggested that Cry5B-PFT promotes the cell-autonomous expression of *prmt-7* in the intestinal cells of *C. elegans*.

In addition, according to the protein sequence alignment of human PRMT7 (HsPRMT7), mouse PRMT7 (MmPRMT7), and *C. elegans* PRMT-7 (CePRMT-7), the amino acids aspartate 143 and glutamate 145 were predicted to be the key catalytic residues of CePRMT-7 (Fig. S1K) [31]. To further examine the importance of PRMT-7 activity in HLH-30 nuclear localization, we generated *app-1*_*p*_*::prmt-7* WT and *app-1*_*p*_*::prmt-7*^*D143A,E145A*^ transgenic animals, which specifically expressed PRMT-7 driven by the intestine-specific promoter, *app-1*_*p*_, in intestinal cells. Expression analyses demonstrated that *prmt-7* WT and *prmt-7*^*D143A,E145A*^ were expressed at similar levels in these transgenic strains (Fig. S1L-N). Moreover, our data showed that the complementation of intestinal PRMT-7 WT significantly rescued the Cry5B-induced nuclear localization of HLH-30 in the *prmt-7(tm4890)* mutant (Figure 1L,M). In contrast, PRMT-7^*D143A,E145A*^, presumably without enzymatic activity, failed to rescue the HLH-30 nuclear localization induced by PFT. These data demonstrated that the enzymatic activity of PRMT-7 is indispensable cell-autonomously for the PFT-elicited HLH-30 nuclear localization in intestinal cells. Taken all together, our findings suggested that PRMT-7 is activated and functions cell-autonomously in the intestine to promote HLH-30 nuclear localization in response to Cry5B-PFT intoxication in *C. elegans*.

### PRMT-7 is required for the PFT-elicited autophagy and plasma membrane pore-repair in the intestinal cells of *C. elegans*

**Figure 2.**
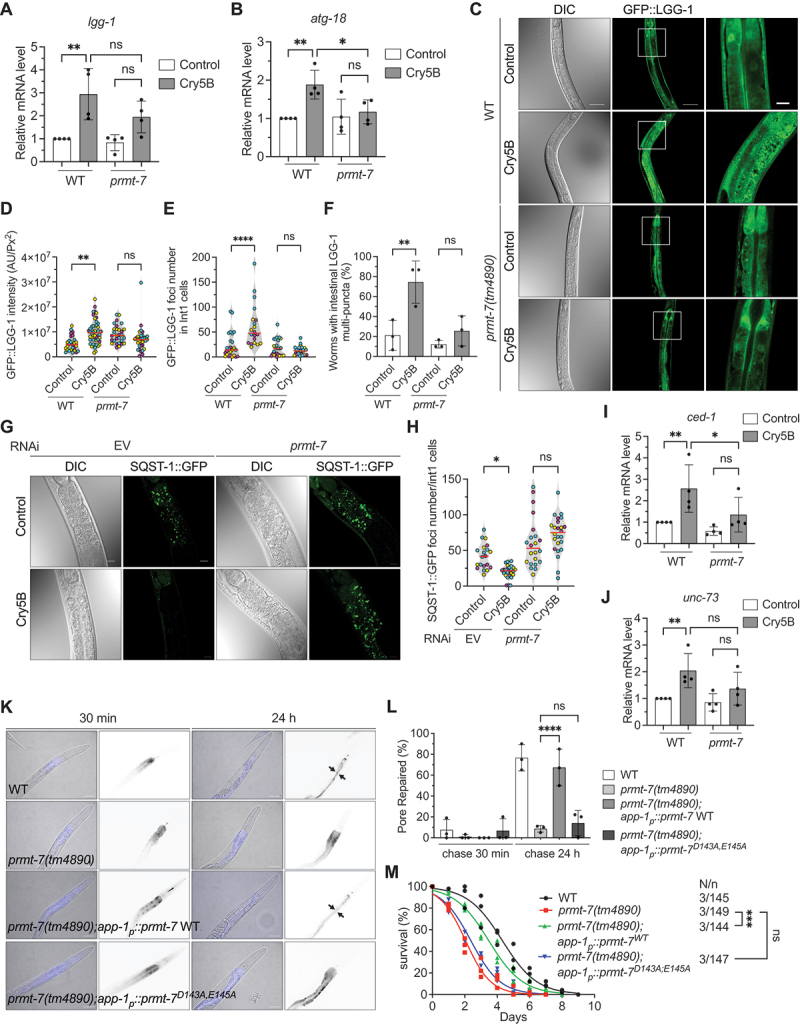
PRMT-7 promotes autophagy and membrane pore-repair to protect *C. elegans* from Cry5B intoxication *in vivo*. (A-B) the qRT-PCR analysis of *lgg-1* (*n* = 12, *N* = 4) and *atg-18* (*n* = 12, *N* = 4) mRNA in N2 wild-type and F×4890*prmt-7(tm4890)* animals fed with Cry5B or control for 3 h. (C) the representative DIC and confocal images of GFP:LGG-1 of the DA2123 wild-type (WT) and YQ349 *prmt-7(tm4890)* mutant fed with Cry5B or control for 3 h. The enlarged images (right panels) are from the frame on the middle panel images of GFP:LGG-1. Scale bars: 50 μm in the left and middle panels; 10 μm in the right panels. (D) the quantification of GFP:LGG-1 intensity in the WT and YQ349 *prmt-7(tm4890)* mutant fed with Cry5B or control for 3 h (*n* = 27 in WT-control, *n* = 50 in WT-Cry5B, *n* = 29 in *prmt-7(tm4890)*-control, *n* = 33 in *prmt-7(tm4890)*-Cry5B). (E) the number of GFP:LGG-1 foci in Int1 cells of the WT and YQ349 *prmt-7(tm4890)* mutant fed with Cry5B or control for 3 h (*n* = 25 in WT-control, *n* = 20 in WT-Cry5B, *n* = 25 in *prmt-7(tm4890)*-control, *n* = 27 in *prmt-7(tm4890)*-Cry5B). (F) the percentage of worms with intestinal GFP:LGG-1 multi-puncta in the WT and YQ349 *prmt-7(tm4890)* groups fed with Cry5B or control for 3 h (*n* = 90, *N* = 3 in WT-control, *n* = 90, *N* = 3 in WT-Cry5B, *n* = 90, *N* = 3 in *prmt-7(tm4890)*-control, *n* = 90, *N* = 3 in *prmt-7(tm4890)*-Cry5B). (G) the representative images of SQST-1:GFP in EV and *prmt-7* knockdown animals fed with Cry5B or control for 3 h. EV indicates the RNAi empty vector L4440. Scale bars: 10 μm. (H) the number of SQST-1:GFP foci in Int1 cells of EV and *prmt-7* knockdown animals fed with control or Cry5B for 3 h (*n* = 20 in EV-control, *n* = 26 in EV-Cry5B, *n* = 22 in *prmt-7-*control, and *n* = 24 in *prmt-7-*Cry5B). EV indicates the RNAi empty vector. (I-J) the qRT-PCR analysis of *ced-1* (*n* = 12, *N* = 4) and *unc-73* (*n* = 12, *N* = 4) mRNA in the N2 WT and F×4890*prmt-7(tm4890)* mutant animals fed with Cry5B or control for 3 h. (K) the representative images of the pore-repair assay. The blue signal indicates the distribution of SYTOX blue in the WT, YQ340 *prmt-7(tm4890)*, YQ483 *prmt-7(tm4890);app-1*_*p*_*::prmt-7* WT, and YQ482 *prmt-7(tm4890);app-1*_*p*_*::prmt-7*^*D143AE145A*^ animals fed with Cry5B for 30 min and transferred to non-Cry5B plates for another 30 min or 24 h for recovery. Arrows indicate the worms with the SYTOX blue signals exclusively in the intestinal lumen. Scale bars: 50 μm. (L) the percentage of pore-repaired animals in the WT (*n* = 90, *N* = 3), YQ340 *prmt-7(tm4890)* (*n* = 90, *N* = 3), YQ483 *prmt-7(tm4890);app-1*_*p*_*::prmt-7* WT (*n* = 90, *N* = 3), and YQ482 *prmt-7(tm4890);app-1*_*p*_*::prmt-7*^*D143AE145A*^ (*n* = 90, *N* = 3) after 30 min or 24 h recovery from Cry5B perforation. (M) the percentage survival of the N2 wild-type (*n* = 145, *N* = 3), F×4890*prmt-7(tm4890)* (*n* = 149, *N* = 3), YQ483 *prmt-7(tm4890);app-1*_*p*_*::prmt-7* WT (*n* = 144, *N* = 3), and YQ482 *prmt-7(tm4890);app-1*_*p*_*::prmt-7*^*D143AE145A*^ (*n* = 147, *N* = 3) animals fed with Cry5B. Data information: all data statistics except fig. 2 M based on: *p < 0.05, **p < 0.01, and ****p < 0.0001 by two-way ANOVA. Fig. 2 M statistics based on: ***p < 0.001 by log-rank test. ns represents non-significance. Means are shown in red lines. Each data set of an independent biological repeat was represented by a different color. See also fig. S2. Source data are available online for this figure.

We previously demonstrated that the nuclear localization of HLH-30, induced by Cry5B-PFT, is required for the expression of autophagic as well as pore-repair genes to eliminate the toxic PFT protein and reconstruct intestinal plasma membrane integrity [5]. As the results shown above, PRMT-7 is indispensable for the full HLH-30 nuclear localization induced by Cry5B; we therefore hypothesized that PRMT-7 is also important for the HLH-30-dependent intrinsic cellular defense (INCED) against Cry5B intoxication *in vivo*. To confirm the INCED function of PRMT-7 in response to PFT intoxication, we first analyzed the mRNA expression level of the previously identified HLH-30-dependent Cry5B-activated key autophagic genes, including *lgg-1* and *atg-18* [5]. Our data demonstrated that the expressions of *lgg-1* and *atg-18* were significantly induced by Cry5B in WT animals, however the expressions of *lgg-1* and *atg-18* in the *prmt-7(tm4890)* mutant were not statistically upregulated upon Cry5B intoxication (Figure 2A,B, respectively). These results suggested that *prmt-7* is also a key regulator for the HLH-30-dependent autophagy induced by PFT. Intriguingly, the expression of *lgg-1* induced by Cry5B, although showed a clear trend of decrease in the *prmt-7(tm4890)* mutant, was not statistically significant compared to WT animals treated with Cry5B. As knockout of *prmt-7* cannot totally abolish the nuclear localization (activity) of HLH-30 upon Cry5B-PFT intoxication (as shown in Figure 1E), our results suggested the expression of *lgg-1* induced by Cry5B-PFT may also be regulated by a, yet unidentified, *prmt-7*-independent mechanism. Nevertheless, to monitor the activation of cellular autophagy induced by Cry5B, we next quantified the GFP:LGG-1 expression level (intensity), the GFP:LGG-1 cellular foci number (a biomarker for autophagosome formation), and the percentage of worms with LGG-1 multicellular puncta in the transgenic *C. elegans* strain, carrying the *lgg-1*_*p*_*::GFP:lgg-1* reporter. Our data showed that all these autophagic indexes were significantly upregulated in wild-type animals by Cry5B but significantly inhibited by the *prmt-7* loss-of-function mutation (Figure 2C–F). Moreover, we also observed a clear trend of the increase of GFP:LGG-1 intensity in the *prmt-7(tm4890)* mutant in control condition, suggesting the autophagic flux in impaired in the in the *prmt-7* mutant animals. Thus, to monitor autophagic flux, we fed SQST-1:GFP transgenic animals with Cry5B. SQST-1/SQSTM1/p62 is a biomarker of autophagic flux that is degraded along with cargo in the autolysosome during autophagy activation [32]. Our data indicated that the degradation SQST-1:GFP induced by Cry5B was significantly inhibited in *prmt-7* knockdown animals (Figure 2G,H). These results together suggested that *prmt-7* also participates in the HLH-30-dependent autophagy activation upon Cry5B-PFT intoxication.

In addition, instantaneous pore-repair is essential for the host to recover from PFT intoxication. The activations of the mRNA expression of the previously identified HLH-30-dependent Cry5B-activated pore-repair genes, *ced-1* and *unc-73* [5], by Cry5B were significantly abolished in the *prmt-7(tm4890)* mutant (Figure 2I,J, respectively). We further utilized the pore-repair assay to monitor the intestinal plasm membrane integrity (PMI) of wild-type, *prmt-7(tm4890)*, *prmt-7(tm4890);app-1*_*p*_*::prmt-7* WT, and *prmt-7(tm4890);app-1*_*p*_*::prmt-7*^*D143A,E145A*^ worms by the membrane impermeable dyes, such as SYTOX blue or propidium iodine (PI) which are smaller in size than the pore created by bacterial PFTs (Fig. S2A) [5,6]. After exposing these four groups of animals to Cry5B for 30-min and chasing them briefly on non-Cry5B plates for another 30 min, SYTOX blue (Figure 2K,L) and PI (Fig. S2B and S2C) were able to leak through the membrane pores generated by Cry5B-PFT from the intestinal lumen into the cytosol of intestinal cells in all Cry5B-treated groups. However, after 24 h of recovery on non-Cry5B plates, SYTOX blue and PI signals in the wild-type and *prmt-7(tm4890);app-1*_*p*_*::prmt-7* WT worms were mostly confined in the intestinal lumen, which suggested that the membrane damages induced by Cry5B-PFT were repaired by INCED systems in these two strains. However, *prmt-7(tm4890)* and *prmt-7(tm4890);app-1*_*p*_*::prmt-7*^*D143A,E145A*^ worms showed diffused SYTOX blue or PI signals in the cytoplasm of intestinal cells. These data not only suggested that the INCED systems were significantly abolished in the *prmt-7* loss-of-function mutants but also demonstrated that the enzymatic activity of PRMT-7 is required for intrinsic membrane repair against Cry5B perforation.

We next tested whether PRMT-7 protects *C. elegans* animals from Cry5B killing *in vivo*. Compared to the wild-type, *prmt-7(tm4890)* animals were significantly hypersusceptible to Cry5B intoxication (Figure 2M). Intestine-specific *prmt-7* WT complementation significantly rescued the survival of the *prmt-7(tm4890)* mutant, while complementation of the enzyme-dead *prmt-7*^*D143A,E145A*^ cannot rescue the survival of *prmt-7(tm4890)* animals. These results reconfirmed that PRMT-7 functions cell-autonomously in the intestinal cells for INCED against Cry5B intoxication. In summary, PRMT-7, which facilitates HLH-30 nuclear localization, plays a protective role in PFT defense through activation of the HLH-30-dependent INCED, including autophagy and membrane pore-repair systems.

### The PRMT-7-mediated INCED is generally required for bacterial PFT defense in *C. elegans*

**Figure 3.**
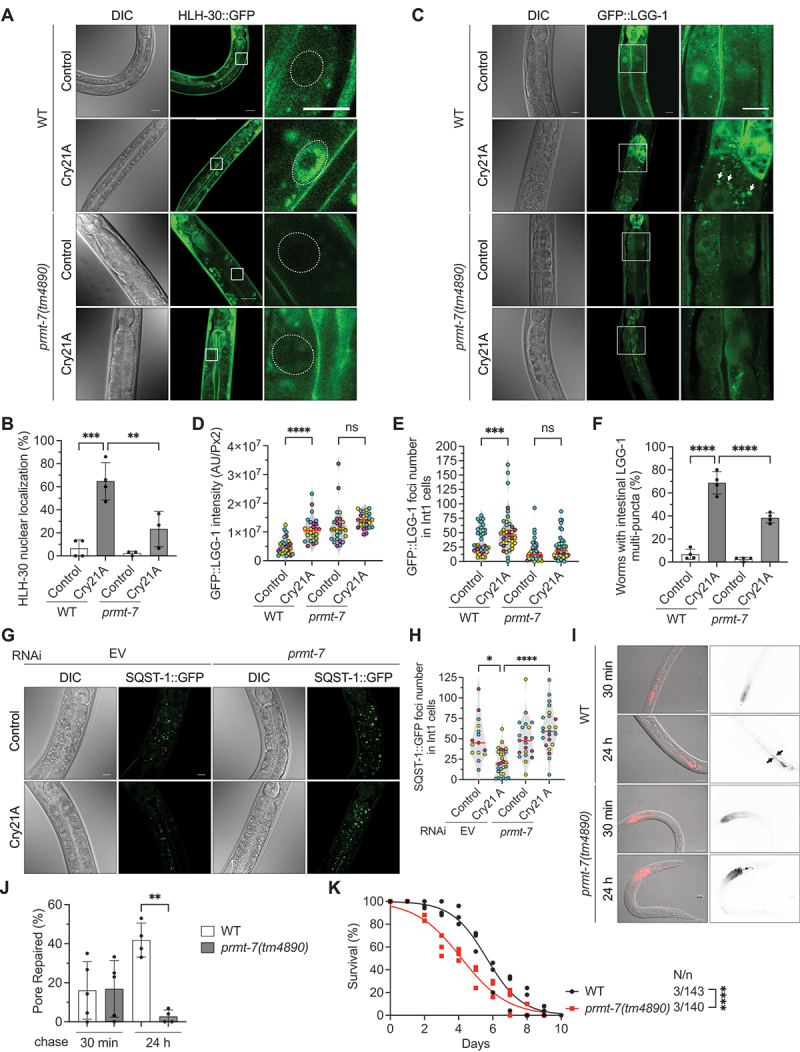
PRMT-7 is also involved in Cry21A-PFT defense through the activation of HLH-30-dependent INCED systems. (A) the representative DIC and confocal images of HLH-30:GFP in the OP433 wild-type (WT) and YQ340 *prmt-7(tm4890)* animals fed with Cry21A or control for 1 h. Enlarged images (right panels) are from the frame on the middle panel images of HLH-30:GFP. The dotted circles represent nuclear areas in the enlarged images. Scale bars: 10 μm. (B) the percentage of worms with the HLH-30 nuclear localization signals in the OP433 WT and YQ340 *prmt-7(tm4890)* groups fed with Cry21A or control for 1 h (*n* = 120, *N* = 4 in WT-control, *n* = 120, *N* = 4 in WT-Cry21A, *n* = 90, *N* = 3 in *prmt-7(tm4890)*-control, *n* = 90, *N* = 3 in *prmt-7(tm4890)*-Cry21A). (C) the representative DIC and confocal images of GFP:LGG-1 in the WT and YQ349 *prmt-7(tm4890)* animals fed with Cry21A or control for 3 h. The enlarged images (right panels) are from the frame on the middle panel images of GFP:LGG-1. The white arrows indicate GFP:LGG-1 cellular puncta in the enlarged images. Scale bars: 10 μm. (D) the GFP:LGG-1 intensity in the WT and YQ349 *prmt-7(tm4890)* animals fed with Cry21A for 3 h (*n* = 26 in WT-control, *n* = 26 in WT-Cry21A, *n* = 26 in *prmt-7(tm4890)*-control, *n* = 26 in *prmt-7(tm4890)*-Cry21A). (E) the number of GFP:LGG-1 foci in Int1 cells in the WT and YQ349 *prmt-7(tm4890)* animals fed with Cry21A or control for 3 h (*n* = 38 in WT-control, *n* = 37 in WT-Cry21A, *n* = 37 in *prmt-7(tm4890)*-control, *n* = 44 in *prmt-7(tm4890)*-Cry21A). (F) the percentage of worms with intestinal GFP:LGG-1 multi-puncta in the WT and YQ349 *prmt-7(tm4890)* groups fed with Cry21A or control for 3 h (*n* = 120, *N* = 4 in WT-control, *n* = 120, *N* = 4 in WT-Cry21A, *n* = 120, *N* = 4 in *prmt-7(tm4890)*-control, *n* = 120, *N* = 4 in *prmt-7(tm4890)*-Cry21A). (G) the representative DIC and confocal images of SQST-1:GFP in EV and *prmt-7* knockdown animals fed with Cry21A or control for 3 h. EV indicates the RNAi empty vector L4440. Scale bars: 10 μm. (H) the number of SQST-1:GFP foci in Int1 cells of EV and *prmt-7* knockdown animals fed with Cry21A or control for 3 h (*n* = 15 in WT-control, *n* = 29 in WT-Cry21A, *n* = 21 in *prmt-7(tm4890)*-control, *n* = 23 in *prmt-7(tm4890)*-Cry21A). EV indicates the RNAi empty vector. (I) the representative images of the pore-repair assay. The red signals indicate the propidium iodine (PI) distribution in the N2 WT and F×4890*prmt-7(tm4890)* animals fed with Cry21A for 30 min and transferred to non-Cry21A plates for another 30 min or 24 h for recovery. Arrows indicate the worms with the PI signals exclusively in the intestinal lumen. Scale bars: 50 μm. (J) the percentage of pore-repaired animals in the N2 WT and F×4890*prmt-7(tm4890)* groups after 30 min or 24 h recovery from Cry21A intoxication (*n* = 150, *N* = 5 in WT-control, *n* = 150, *N* = 5 in WT-Cry21A, *n* = 120, *N* = 4 in *prmt-7(tm4890)*-control, *n* = 120, *N* = 4 in *prmt-7(tm4890)*-Cry21A). (K) the percentage survival of the WT (*n* = 143, *N* = 3) and *prmt-7(tm4890)* (*n* = 140, *N* = 3) animals fed with Cry21A. Data information: all data statistics except fig. 3K based on: *p < 0.05, **p < 0.01, ***p < 0.001 and ****p < 0.0001 by two-way ANOVA. Fig. 3K statistics based on: ****p < 0.0001 by log-rank test. ns represents non-significance. Means are shown in red lines. Each data set of an independent biological repeat was represented by a different color. See also fig. S3. Source data are available online for this figure.

To test whether the PRMT-7-dependent HLH-30 nuclear transactivation is generally required for the INCED against bacterial pore-forming toxins, we first fed *C. elegans* with the other two different PFTs, Cry21A and hemolysin Ahh1. Cry21A from *Bacillus thuringiensis* and hemolysin Ahh1 from *Aeromonas dhakensis* also perforate the plasma membrane of intestinal cells via different receptors than Cry5B to intoxicate *C. elegans* [4,33]. Our data showed that both Cry21A and hemolysin Ahh1 significantly induced HLH-30 nuclear localization in wild-type animals, while the *prmt-7* loss-of-function mutation significantly abolished these phenomena (Figure 3A,B, S3A, and S3B). Together, our results reconfirm that bacterial PFTs elicit nuclear localization of HLH-30 in their target cells in a PRMT-7-dependent manner.

Next, we proceeded to reconfirm the role of PRMT-7 in INCED against Cry21A. Our results showed that the reporters for autophagosome formation, including GFP:LGG-1 intensity, GFP:LGG-1 foci number in Int1 cells, and the percentage of animals with GFP:LGG-1 multi-puncta were all statistically downregulated in the *prmt-7(tm4890)* mutant compared to wild-type animals in response to Cry21A intoxication (Figure 3C–F). Concomitantly, *prmt-7* knockdown significantly inhibited the degradation of SQST-1:GFP induced by Cry21A, suggesting that the autophagic flux was interrupted (Figure 3G,H).

In addition, in the pore-repair activity analysis, *C. elegans* animals fed with Cry21A for 30 min and then recovered on non-Cry21A plates for 30 min showed a diffused propidium iodine (PI) signal in the cytosol of intestinal cells, reconfirming that Cry21A also generated pores in intestinal cells (Figure 3I,J). After 24 h, N2 wild-type animals repaired the membrane damage as the PI signals were distributed exclusively in the intestinal lumen. However, *prmt-7(tm4890)* animals failed to seal the intestinal plasma membrane pores generated by Cry21A. Moreover, *prmt-7(tm4890)* mutant worms were significantly hypersusceptible to Cry21A killing compared to the N2 wild-type (Figure 3K). Overall, our findings from Cry5B and Cry21A produced by *B. thuringiensis* and Ahh1 secreted by human-pathogenic *A. dhakensis* demonstrated that PRMT-7 is generally required for the activation of HLH-30 triggered by bacterial PFTs in *C. elegans*.

### PRMT-7 interacts with HLH-30 in the cytosol and promotes HLH-30 nuclear translocation in response to PFT intoxication

**Figure 4.**
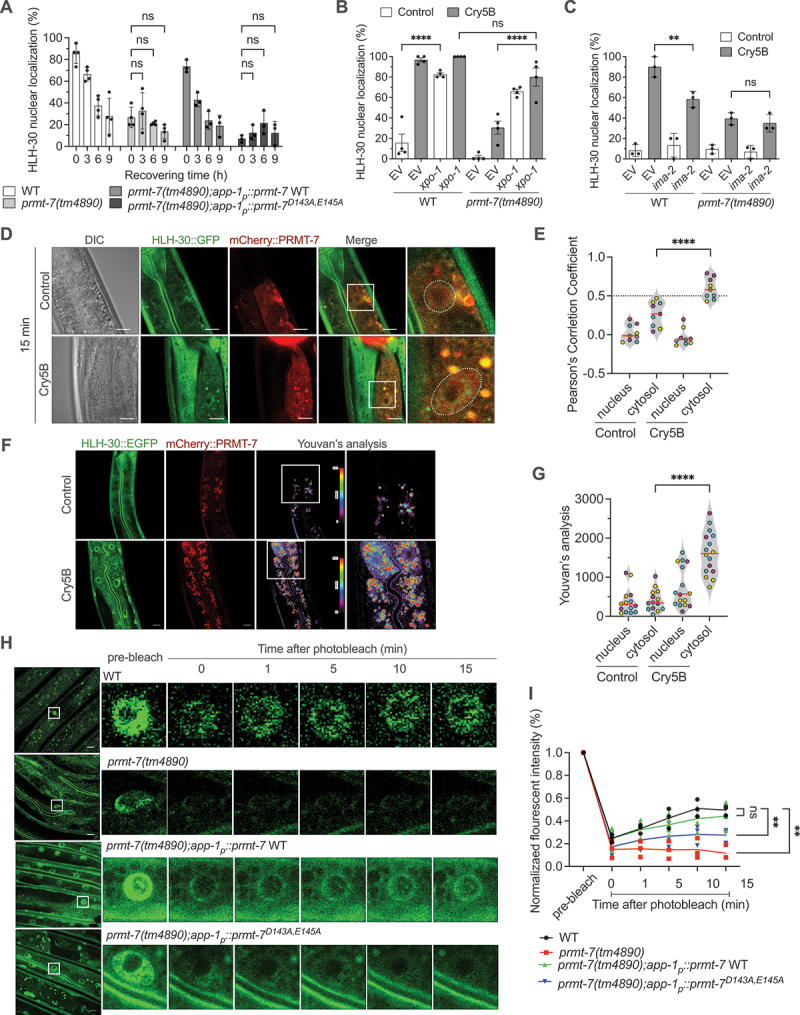
PRMT-7 functions in the cytosol to promote HLH-30 nuclear translocation in response to Cry5B intoxication. (A) the percentage of worms with HLH-30 nuclear localization in the WT (*n* = 120, *N* = 4), YQ340 *prmt-7(tm4890)* (*n* = 120, *N* = 4), YQ483 *prmt-7(tm4890);app-1*_*p*_*::prmt-7* WT (*n* = 90, *N* = 3) and YQ482 *prmt-7(tm4890);app-1*_*p*_*::prmt-7*^*D143A,E145A*^ (*n* = 90, *N* = 3) animals fed with Cry5B for 1 h and then transferred to non-Cry5B plates for 0, 3, 6, 9 h. (B) the percentage of animals with HLH-30 nuclear localization in EV and *xpo-1* RNAi knockdown WT (*n* = 120, *N* = 4) and YQ340 *prmt-7(tm4890)* (*n* = 120, *N* = 4) animals fed with Cry5B for 1 h. EV indicates the RNAi empty vector L4440. (C) the percentage of worms with HLH-30 nuclear localization in EV and *ima-2* RNAi knockdown WT (*n* = 90, *N* = 3) and *prmt-7(tm4890)* (*n* = 90, *N* = 3) animals fed with Cry5B for 1 h. EV indicates the RNAi empty vector L4440. (D) the representative confocal images of HLH-30:GFP and mCherry:PRMT-7 in YQ454 *hlh-30:GFP;mCherry:prmt-7* animals fed with control or Cry5B for 15 min. Scale bar: 10 μm. (E) the Pearson’s correlation coefficient of HLH-30:GFP and mCherry:PRMT-7 in the nucleus or cytosol of YQ454 *hlh-30:GFP;mCherry:prmt-7* animals fed with control or Cry5B after 15 min (*n =* 9 in control-nucleus, *n =* 9 in control-cytosol, *n =* 9 in Cry5B-nucleus, and *n =* 9 in Cry5B-cytosol). Pearson’s correlation coefficient: −1 to 0 indicates negative correlation, 0 to 0.5 indicates no correlation, and 0.5 to 1 indicates positive correlation. (F) the representative images of fluorescence resonance energy transfer (FRET) of HLH-30:EGFP and mCherry:PRMT-7 in YQ454 *hlh-30:EGFP;mCherry:prmt-7* animals fed with Cry5B for 1 h. Scale bar: 10 μm. (G) the quantification of FRET signals between HLH-30:EGFP and mCherry:PRMT-7 in nucleus or cytosol by Youvan’s analysis (*n =* 14, *N* = 3 in control-nucleus, *n =* 14, *N* = 3 in control-cytosol, *n =* 16, *N* = 3 in Cry5B-nucleus, and *n =* 16, *N* = 3 in cry5B-cytosol). The YQ454 *hlh-30:EGFP;mCherry:prmt-7* animals were exposed to Cry5B for 1 h. (H) the representative images of fluorescence recovery after photobleaching (FRAP) of nuclear HLH-30:GFP in the WT, YQ340 *prmt-7(tm4890)*, YQ483 *prmt-7(tm4890);app-1*_*p*_*::prmt-7* WT and YQ482 *prmt-7(tm4890);app-1*_*p*_*::prmt-7*^*D143A,E145A*^ animals exposed to Cry5B for 30 min. Scale bar: 10 μm. (I) the quantification of nuclear HLH-30:GFP intensity of the WT (*N* = 3), YQ340 *prmt-7(tm4890)* (*N* = 3), YQ483 *prmt-7(tm4890);app-1*_*p*_*::prmt-7* WT (*N* = 5), and YQ482 *prmt-7(tm4890);app-1*_*p*_*::prmt-7*^*D143A,E145A*^ (*N* = 3) animals exposed to Cry5B for 30 min. HLH-30:GFP intensity is normalized with the pre-bleached HLH-30:GFP signal. Data information: all data statistics based on: **p < 0.01 and ****p < 0.0001 by two-way ANOVA. ns represents non-significance. Means are shown in red lines. Each data set of an independent biological repeat was represented by a different color. See also fig. S4. Source data are available online for this figure.

We next attempted to investigate the molecular mechanism underlying the PRMT-7-dependent nuclear translocation of HLH-30 upon PFT intoxication. To this end, we first designed a pulse-chase assay in which we fed HLH-30:GFP worms with Cry5B for 1 h and then transferred the animals to normal *C. elegans* culture plates (without Cry5B toxin) for another 9 h to monitor the dynamics of the HLH-30 cellular distribution (Figure 4A and S4A). After 1 h of Cry5B exposure, the nuclear HLH-30:GFP signal reached to the plateau and gradually decreased over time in the wild-type animals without further Cry5B-PFT stimulation, suggesting an intrinsic control mechanism of the HLH-30 homeostasis in this dynamic cellular process. However, the dynamics of HLH-30:GFP redistribution in response to PFT intoxication was diminished in the *prmt-7(tm4890)* mutant. Moreover, the intestinal complementation of PRMT-7 WT rescued the dynamics of cellular distribution of HLH-30:GFP in response to Cry5B intoxication, while animals with the complementation of enzyme-dead PRMT-7^D143A,E145A^ could not regulate the dynamics of HLH-30:GFP localization. To eliminate the possibility that only one-h Cry5B exposure was not sufficient to sustain the HLH-30 nuclear location, we quantified the percentage of nuclear HLH-30:GFP in the animals continuously exposed to Cry5B for 1, 4, 7 and 10 h (Fig. S4A and S4B). Consistently, our data showed that the dynamics of HLH-30:GFP cellular location after Cry5B intoxication is maintained in wild-type and *prmt-7(tm4890);app-1*_*p*_*::prmt-7* WT animals, but Cry5B-PFT failed to evoke the dynamics of HLH-30:GFP nuclear localization in *prmt-7(tm4890)* and *prmt-7(tm4890);app-1*_*p*_*::prmt-7*^*D143A,E145A*^ animals. Together, our results implied that intestinal PRMT-7 is required cell-autonomously for the dynamics of HLH-30 shuttling between nucleus and cytosol in response to PFT intoxication.

Except for the aforementioned RRAG GTPase-MTORC1-YWHA/14-3-3 system in the cytosol, the nuclear-cytoplasmic shuttling of HLH-30/TFEB can also be regulated by the nuclear XPO (exportin) [34,35] as well as the nuclear IMA/IPO (importin) [13]. To test whether exportin or importin participates in the PRMT-7-dependent HLH-30 nuclear translocation induced by Cry5B-PFT, we first knocked down *xpo-1*, which encodes an exportin in *C. elegans*, in wild-type and *prmt-7(tm4890)* animals and fed them with Cry5B. Our data showed that *xpo-1* knockdown can significantly enhance HLH-30 nuclear localization in wild-type animals even without Cry5B treatment (Figure 4B). Moreover, knockdown of *xpo-1* can further enhance the nuclear localization of HLH-30 in the *prmt-7(tm4890)* animals treated with Cry5B. These genetic epistasis analyses suggested a notion that XPO-1 May act downstream of PRMT-7 to regulate the exportation of HLH-30 after transferring into the nucleus upon Cry5B intoxication. Intriguingly, knockdown of *ima-2*, encodes an importin, can significantly suppress the nuclear localization of HLH-30 induced by Cry5B in wild-type animals, but cannot further suppress it in *prmt-7(tm4890)* animals (Figure 4C). These genetic epistasis data demonstrated that *prmt-7* and *ima-2* may function in the same genetic pathway to regulate nuclear translocation of HLH-30 from the cytosol during Cry5B-PFT intoxication. Taken all together, our data reconfirmed the role of PRMT-7 in regulating the cytoplasmic-nuclear shuttling dynamics of HLH-30 upon Cry5B-PFT intoxication.

Given that PRMT-7 proteins are both expressed in the cytoplasm and nucleus, we hypothesized that PRMT-7 May function either in the cytosol to promote HLH-30 nuclear translocation or in the nucleus to prevent HLH-30 exportation upon Cry5B intoxication. First, we aimed to monitor the potential *in vivo* interaction between HLH-30 and PRMT-7 in the intestinal cells of *C. elegans*. To this end, we injected an *app-1*_*p*_*::mCherry:prmt-7* transgene into the HLH-30:GFP animal and obtained a mosaic mCherry:PRMT-7 expression line. The *hlh-30:GFP;mCherry:prmt-7* worms were fed with Cry5B or control for 5, 10, and 15 min and analyzed by confocal microscopy (Figure 4D,E, S4C, and S4D). The correlations between HLH-30:GFP and mCherry:PRMT-7 signals were calculated by Pearson’s correlation coefficient analysis. Our results demonstrated a positive correlation (Pearson’s correlation coefficient > 0.5) between HLH-30:GFP and mCherry:PRMT-7 in the cytosol after Cry5B treatment for 5, 10 and 15 min, respectively, which suggested that the colocalization of HLH-30:GFP and mCherry:PRMT-7 was significantly increased in the cytosol, but not in the nucleus, of intestinal cells upon PFT intoxication. Moreover, the nuclear PRMT-7 level in the intestinal cells was not altered by Cry5B-PFT (Fig. S4E and S4F), strengthening the possibility of the cytosolic role of PRMT-7 in regulating the cytoplasmic-nuclear shuttling dynamics of HLH-30 in response to PFT intoxication. Next, we also applied Förster/fluorescence resonance energy transfer (FRET) analysis to determine the protein-protein interaction between HLH-30:EGFP and mCherry:PRMT-7 *in vivo*. Compared to the control group, Cry5B-treated animals showed a robust sensitization in mCherry:PRMT-7, the acceptor protein, indicating the energy transfer from EGFP to mCherry in the cytosol of intestinal cells induced by Cry5B-PFT (Figure 4F,G). The proximity of HLH-30:EGFP and mCherry:PRMT-7 implied an increase in physical interaction between these two proteins in the cytosol of intestinal cells upon Cry5B intoxication *in vivo*. Together, these data suggested a notion that Cry5B-PFT stimulates the interaction between PRMT-7 and HLH-30 in the cytosol to facilitate the subsequent HLH-30 nuclear translocation in response to PFT intoxication *in vivo*.

Finally, to reconfirm the role of PRMT-7 in regulating the cytoplasmic-nuclear shuttling dynamics of HLH-30, we performed a fluorescence recovery after photobleaching (FRAP) analysis on the nuclear HLH-30:GFP signals in wild-type and *prmt-7(tm4890)* animals (Figure 4H,I). Our results showed that the nuclear HLH-30:GFP signals were gradually restored over time after photobleaching in the wild-type and *prmt-7(tm4890);app-1*_*p*_*::prmt-7* WT animals treated with Cry5B. However, the dynamics of the Cry5B-induced HLH-30 nuclear localization signal was completely abolished in the *prmt-7(tm4890)* and *prmt-7(tm4890);app-1*_*p*_*::prmt-7*^*D143A,E145A*^ mutants. These results suggested that PRMT-7 is essential for the Cry5B-PFT-induced nuclear translocation of HLH-30 from the cytoplasm. In sum, our results suggested that PRMT-7 May interact with HLH-30 and function in the cytosol to regulate the cytoplasmic-nuclear shuttling dynamics of HLH-30 in response to PFT intoxication.

### PRMT-7 antagonizes the RRAG GTPases-MTORC1-YWHA/14-3-3 system to promote HLH-30 nuclear translocation

**Figure 5.**
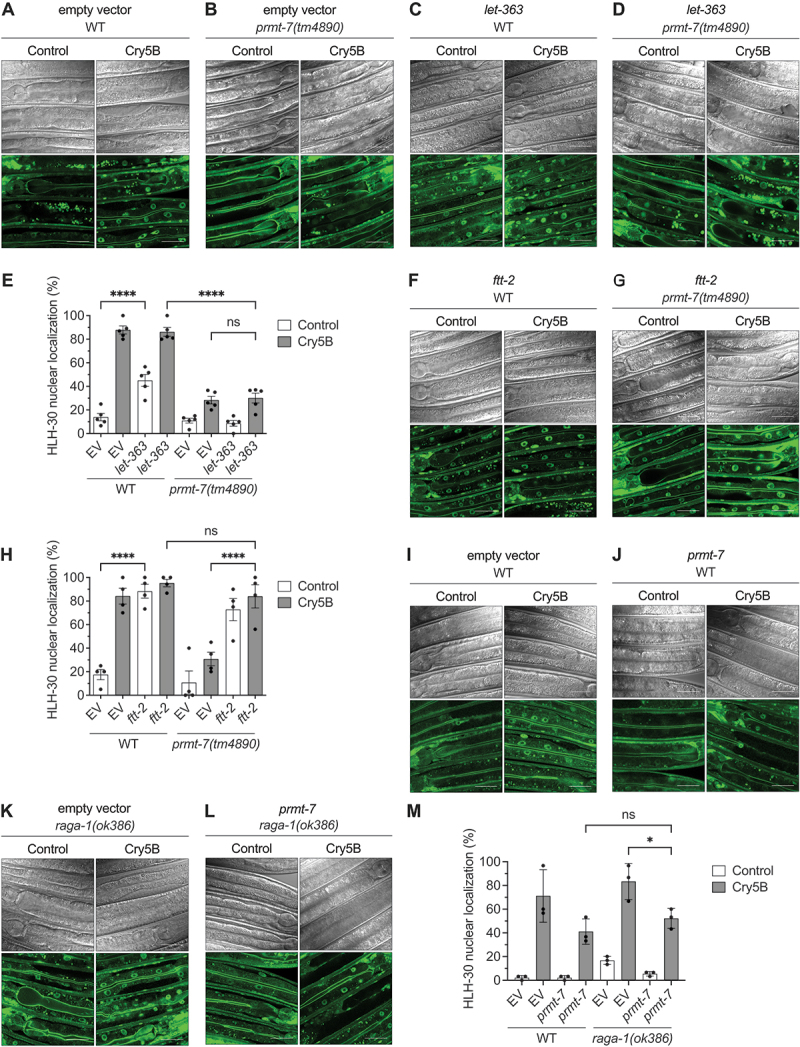
*Prmt-7* promotes HLH-30 nuclear translocation by interfering with the RRAG GTPases-MTORC1-YWHA/14-3-3 system. (A-D) the representative images of the wild-type (WT) and YQ340 *prmt-7(tm4890)* animals knocked-down by EV and *let-363* RNAi and fed with Cry5B or control for 1 h. EV indicates the RNAi empty vector L4440. Scale bar: 30 μm. (E) the percentage of worms with HLH-30 nuclear localization in EV and *let-363* RNAi knockdown WT (*n* = 150, *N* = 5) and YQ340 *prmt-7(tm4890)* (*n* = 150, *N* = 5) animals fed with Cry5B or control for 1 h. EV indicates the RNAi empty vector L4440. (F-G) the representative images of *ftt-2* RNAi knocked-down WT and YQ340 *prmt-7(tm4890)* animals fed with Cry5B or control for 1 h. EV indicates the RNAi empty vector L4440. Scale bar: 30 μm. (H) the percentage of worms with HLH-30 nuclear localization in EV and *ftt-2* RNAi knockdown WT (*n* = 120, *N* = 4) and YQ340 *prmt-7(tm4890)* (*n* = 120, *N* = 4) animals fed with Cry5B or control for 1 h. EV indicates the RNAi empty vector L4440. (I-L) the representative images of EV and *prmt-7* RNAi knockdown WT and YQ534 *raga-1(ok386)* animals fed with Cry5B or control for 1 h. EV indicates the RNAi empty vector L4440. Scale bar: 30 μm. (M) the percentage of worms with HLH-30 nuclear localization in EV and *prmt-7* RNAi knockdown WT (*n* = 90, *N* = 3) and YQ534 *raga-1(ok386)* (*n* = 90, *N* = 3) animals fed with Cry5B or control for 1 h. EV indicates the RNAi empty vector L4440. Data information: all data statistics based on: *p < 0.05 and ****p < 0.0001 by two-way ANOVA. ns represents non-significance. Source data are available online for this figure.

Given that the above data suggested a cytosolic role for PRMT-7 in the regulation of HLH-30 nuclear shuttling, we next tested whether PRMT-7 participates in the well-known cytosolic RRAG GTPases-MTORC1-YWHA/14-3-3 system to regulate the HLH-30 nuclear localization induced by Cry5B-PFT. We first knocked-down *let-363/MTOR* in wild-type and *prmt-7(tm4890)* animals (Figure 5A–E). Our results showed that RNAi of *let-363* in wild-type animals can significantly enhance the nuclear localization of HLH-30 even without Cry5B treatment (Figure 5A,C,E). However, RNAi of *let-363* in *prmt-7(tm4890)* animals cannot rescue the HLH-30 nuclear localization suppressed by the *prmt-7* mutation upon Cry5B treatment (Figure 5B–E). These genetic epistasis data suggested that *prmt-7* functions downstream and in the same genetic/molecular mechanism of the MTOR signal to regulate the HLH-30 nuclear localization upon Cry5B intoxication.

Next, we knocked-down *ftt-2/14-3-3* in wild-type and *prmt-7(tm4890)* animals (Figure 5A,B,F–H). Our results showed that RNAi of *ftt-2* can significantly enhance HLH-30 nuclear localization in wild-type animals without Cry5B treatment and can also further enhance HLH-30 nuclear localization in the *prmt-7(tm4890)* animals treated with Cry5B-PFT. These genetic epistasis analyses suggested that *ftt-2* acts downstream of the *prmt-7* signal to regulate HLH-30 nuclear localization. Taken all together, our genetic analyses suggested that PRMT-7 May function in the cytosol to modulate the MTORC1-YWHA/14-3-3 sequestering system and to promote the Cry5B-induced HLH-30 nuclear translocation.

It has been reported that the RRAG GTPase complex can interact with the N-terminal 40 amino acid residues of TFEB and lead to the MTORC1-dependent phosphorylation and inhibition of TFEB [18,19]. We next examined whether *raga-1/RRAGA* also participates in this PRMT-7-dependent regulation of HLH-30 nuclear localization. To this end, we knocked-down *prmt-7* in wild-type and *raga-1(ok386)* animals (Figure 5I–M). Our results showed that the *raga-1* loss-of-function mutation can significantly enhance HLH-30 nuclear localization even without Cry5B treatment compared to the wild-type. However, the *raga-1* mutation cannot rescue the full HLH-30 nuclear localization suppressed by *prmt-7* RNAi upon Cry5B intoxication. These data suggested that *prmt-7* functions and antagonizes to the *raga-1* signal to regulate HLH-30 nuclear localization induced by Cry5B-PFT. Taken all together, our genetic epistasis analyses suggested a working model that PRMT-7 May function to antagonize the cytosolic RRAG GTPases-MTORC1-YWHA/14-3-3 system and thus promote HLH-30 nuclear localization upon Cry5B intoxication.

### The PRMT-7-dependent methylation of HLH-30 R38 is required for HLH-30 nuclear translocation in response to PFT intoxication

**Figure 6.**
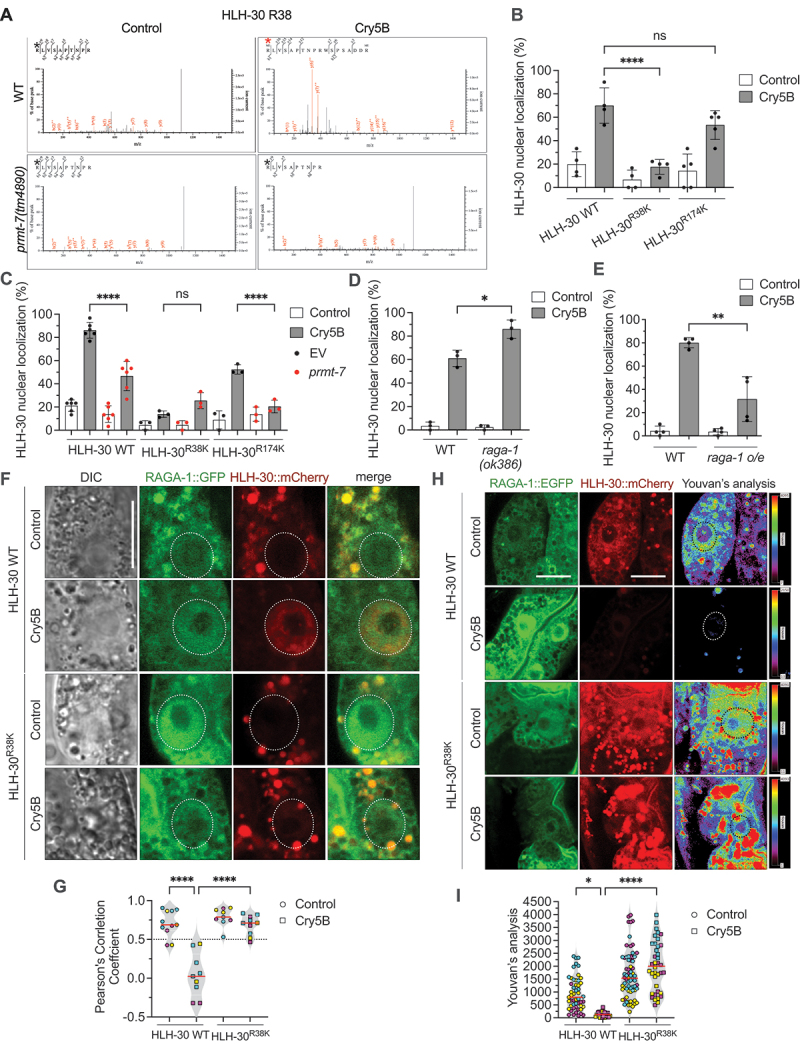
PRMT-7 participates in the methylation of HLH-30 R38 to antagonize the interaction between HLH-30 and RAGA-1 upon Cry5B intoxication. (A) the mass spectrometry results showed the methylation status of HLH-30 R38 in the wild-type (WT) and YQ340 *prmt-7(tm4890)* animals fed with Cry5B or control for 1 h. The black asterisks indicate the non-methylated arginine 38 residues in HLH-30. The red asterisk indicates the methylated arginine 38 residue in HLH-30. (B) the percentage of animals with HLH-30:GFP nuclear localization in the YQ479 *app-1*_*p*_*::hlh-30* WT*::mCherry* (*n* = 120, *N* = 4), YQ554 *app-1*_*p*_*::hlh-30*^*R38K*^*::mCherry* (*n* = 120, *N* = 4) and YQ600 *app-1*_*p*_*::hlh-30*^*R174K*^*::mCherry* (*n* = 150, *N* = 5) transgenic animals fed with Cry5B or control for 1 h. (C) the percentage of EV or *prmt-7* RNAi knockdown animals with HLH-30:GFP nuclear localization in the YQ479 *app-1*_*p*_*::hlh-30* WT*::mCherry* (*n* = 180, *N* = 6), YQ554 *app-1*_*p*_*::hlh-30*^*R38K*^*::mCherry* (*n* = 90, *N* = 3) and YQ600 *app-1*_*p*_*::hlh-30*^*R174K*^*::mCherry* (*n* = 90, *N* = 3) transgenic animals fed with Cry5B or control for 1 h. EV indicates the RNAi empty vector L4440. (D) the percentage of animals with HLH-30:GFP nuclear localization in the WT or YQ534 *raga-1(ok386)* animals fed with Cry5B or control for 30 min (*n* = 90, *N* = 3 in WT-control, *n* = 90, *N* = 3 in WT-Cry5B, *n* = 90, *N* = 3 in *raga-1(ok386)*-control, *n* = 90, *N* = 3 in *raga-1(ok386)*-Cry5B). (E) the percentage of animals with HLH-30:GFP nuclear localization in the WT or YQ565 *raga-1* overexpression (*o/e*) animals fed with Cry5B or control for 1 h (*n* = 120, *N* = 4 in WT-control, *n* = 120, *N* = 4 in WT-Cry5B, *n* = 120, *N* = 4 in *raga-1 o/e*-control, *n* = 120, *N* = 4 in *raga-1 o/e*-Cry5B). (F) the representative confocal images of RAGA-1:EGFP and HLH-30:mCherry signals in HLH-30 WT:mCherry;RAGA-1:EGFP (YQ601) or HLH-30^R38K^::mCherry;RAGA-1:EGFP (YQ596) animals fed with Cry5B or control for 1 h. The dotted circles represent nuclear area. Scale bar: 10 μm. (G) the Pearson’s correlation coefficient analysis of RAGA-1:GFP and HLH-30:mCherry signals in the intestinal cytosol of YQ601 *hlh-30 WT ::mCherry;raga-1:GFP* or YQ596 *hlh-30*^*R38K*^*::mCherry;raga-1:GFP* animals fed with Cry5B or control for 1 h (*n =* 11 in HLH-30 WT-control, *n =* 9 in HLH-30 WT-Cry5B, *n =* 9 in HLH-30^R38K^-control, and *n =* 10 in HLH-30^R38K^-Cry5B). Pearson’s correlation coefficient: −1 to 0 indicates negative correlation, 0 to 0.5 indicates no correlation, and > 0.5 indicates positive correlation. (H) the representative images of fluorescence resonance energy transfer (FRET) analysis of RAGA-1:EGFP and HLH-30:mCherry signals in the YQ601 *hlh-30* WT*::mCherry;raga-1:EGFP* or YQ596 *hlh-30*^*R38K*^*::mCherry;raga-1:EGFP* animals fed with Cry5B or control for 1 h. The dotted circles represent nuclear area. Scale bar: 10 μm. (I) the quantification of the FRET signals between RAGA-1:EGFP and HLH-30:mCherry in the cytosol of intestinal cells in (H) by Youvan’s analysis (*n =* 51 in HLH-30 WT-control, *n =* 26 in HLH-30 WT-Cry5B, *n =* 68 in HLH-30^R38K^-control, and *n =* 38 in HLH-30^R38K^-Cry5B). Data information: all data statistics based on: *p < 0.05, **p < 0.01 and ****p < 0.0001 by two-way ANOVA. ns represents non-significance. Means are shown in red lines. Each data set of an independent biological repeat was represented by a different color. See also figs. S5 and S6. Source data are available online for this figure.

Given that the above data demonstrated that the enzymatic activity of PRMT-7 is indispensable for the HLH-30 nuclear translocation induced by Cry5B-PFT, we next examined whether PRMT-7 May participate in the posttranslational arginine methylation of HLH-30. To this end, we first conducted a liquid chromatograph-tandem mass spectrometry (LC-MS/MS) analysis for HLH-30 proteins extracted from wild-type and *prmt-7(tm4890)* animals fed with Cry5B or control to determine whether some arginine residues of HLH-30 are methylated in a PRMT-7-dependent manner and specifically induced by PFT. We identified several arginine residues that were methylated in HLH-30. Among them, the methylations of arginine 38 and arginine 174 of HLH-30 were specifically induced by Cry5B in wild-type animals, but not in the *prmt-7(tm4890)* mutant (Figures. 6A and S5A). In order to confirm whether the PRMT-7-dependent methylations of HLH-30 R38 and HLH-30 R174 were indispensable to HLH-30 subcellular redistribution, we generated the transgenic *C. elegans* strains expressing HLH-30 WT:mCherry, HLH-30^R38K^::mCherry, or HLH-30^R174K^::mCherry, respectively, in the *hlh-30(tm1978)* mutant background. The arginine/R to lysine/K (arginine methylation-deficient) mutations abolish the PRMT-7-mediated methylation of HLH-30 on these arginine residues. Our results showed that HLH-30 nuclear localization was significantly suppressed only in the HLH-30^R38K^ mutant fed with Cry5B compared to the HLH-30 WT animals, suggesting at least R38, but not R174, is a critical residue that was methylated in a PRMT-7-dependent manner to regulate HLH-30 distribution in response to Cry5B intoxication (Figures. 6B and S5B-S5D). To reconfirm this notion, we knocked down *prmt-7* in HLH-30 WT, HLH-30^R38K^ and HLH-30^R174K^ animals and exposed them to Cry5B or control for 1 h (Figure 6C). Our results showed that, compared to the RNAi control (EV) group, *prmt-7* knockdown could not further downregulate HLH-30 nuclear localization in the HLH-30^R38K^ mutant, indicating that *prmt-7* and the methylation of HLH-30 R38 signal are in the same genetic pathway to modulate the cellular redistribution of HLH-30 upon Cry5B intoxication. In sum, we demonstrated that R38 of HLH-30 is not only a key residue for the PRMT-7-induced methylation but also indispensable to HLH-30 nuclear localization in response to PFT intoxication.

As aforementioned, the subcellular localization of TFEB is tightly regulated by the RRAG GTPases-MTORC1-YWHA/14-3-3 sequester system in the cytosol [19]. In normal conditions, the RRAG GTPase complex binds to the N-terminal RRAG binding domain (1–40 amino acids) of TFEB on the surface of the lysosome and recruits the MTORC1 complex to phosphorylate at serine 211 of TFEB. The phosphorylated TFEB S211 is then sequestered in the cytosol by the YWHA/14-3-3 phospho-binding protein. Intriguingly, we also observed that the phosphorylation signal on HLH-30 S201, which is equivalent to TFEB S211, was reversely corelated with the PRMT-7-dependent HLH-30 R38 methylation signal induced by Cry5B from our LC-MS/MS analyses (Fig. S5E). Moreover, the R38 of HLH-30 is aligned to the R24 of TFEB in the RRAGA-RRAGC binding region, which is reported to be important for their interaction (Fig. S6A and S6B). Taken together, these observations were reminiscent of the notion that PRMT-7 May antagonize the RRAG GTPases-MTORC1-YWHA/14-3-3 sequester system by interrupting the interaction between HLH-30 and the RRAG GTPases/MTORC1 complex to diminish the phosphorylation of HLH-30 S201 and thus prevent cytosolic sequestration by FTT-2/14-3-3 and induce HLH-30 nuclear localization upon Cry5B intoxication.

Therefore, we next aimed to reconfirm the roles of RAGA-1/RRAGA and RAGC-1/RRAGC in Cry5B-induced HLH-30 activation by feeding *raga-1(ok386)* and *ragc-1(tm1974)* mutant animals with Cry5B or control for 30 min (Figures. 6D and S6C). Our results showed that the presumable loss-of-function mutations of *raga-1* and *ragc-1* can significantly enhance the HLH-30 nuclear localization elicited by Cry5B treatment compared to the wild-type. Moreover, the Cry5B-induced HLH-30 nuclear redistribution was significantly suppressed in the *raga-1* overexpression transgenic animals (Figure 6E). We next examined whether the PRMT-7-dependent HLH-30 R38 methylation may affect the interaction between HLH-30 and RAGA-1 *in vivo*. To this end, we generated a transgenic *C. elegans* strain with the expression of both RAGA-1:mCherry and HLH-30:EGFP. We determined the potential *in vivo* interaction between RAGA-1:mCherry and HLH-30:EGFP in EV or *prmt-7* RNAi knockdown animals by confocal fluorescence microscopy and FRET analyses (Fig. S6D-S6G). Our results showed that, according to the Pearson’s correlation coefficient analysis, Cry5B treatment significantly decreased the positive correlation between RAGA-1:mCherry and HLH-30:EGFP signals in the cytosol of intestinal cells, but the correlation between these two signals in the cytosol of intestinal cells in the *prmt-7* knockdown animals were still maintained (Fig. S6D and S6E). Consistently, the FRET analyses demonstrated that cytosolic RAGA-1:mCherry signal, as an acceptor, could not be sensitized by energy from HLH-30:EGFP in worms fed with Cry5B, while energy from HLH-30:EGFP transferred to RAGA-1:mCherry in *prmt-7* knockdown animals fed with both Cry5B and control (Fig. S6F and S6G). These data implied that PRMT-7 facilitates the dissociation between intestinal HLH-30 and RAGA-1 in the cytosol upon Cry5B-FPT intoxication.

To further reconfirm the notion that the PRMT-7-dependent HLH-30 R38 methylation regulates the interaction between HLH-30 and RAGA-1 in the cytosol, we generated the *C. elegans* strains expressing RAGA-1:EGFP in the *hlh-30(tm1978);app-1*_*p*_*::hlh-30* WT*::mCherry* or *hlh-30(tm1978);app-1*_*p*_*::hlh-30*^*R38K*^*::mCherry* backgrounds. Our confocal image analysis showed that the HLH-30^R38K^::mCherry signals can still maintain a significant correlation with RAGA-1:EGFP in the cytosol of intestinal cells under Cry5B treatment compared to the wild-type HLH-30 WT:mCherry (Figure 6F,G). Consistently, our FRET analyses also demonstrated a robust interaction between RAGA-1:EGFP and HLH-30^R38K^::mCherry in the animals even when exposed to Cry5B, while PFT intoxication significantly abolished the FRET signal between RAGA-1:EGFP and HLH-30 WT:mCherry (Figure 6H,I). Together, our results strengthen the notion that the PRMT-7-dependent methylation of HLH-30 R38 is essential for the dissociation between HLH-30 and RAGA-1 induced by Cry5B-PFT. In conclusion, we revealed that the methylation of the specific R38 residue in the Rag complex binding domain of HLH-30 is induced by PFT intoxication in a PRMT-7-dependent manner. Moreover, this PTM of HLH-30 R38 disrupts the binding of HLH-30 with RAGA-1 and therefore promotes HLH-30 nuclear translocation.

### PRMT7 is evolutionarily conserved to regulate TFEB nuclear localization and maintain plasma membrane integrity in human intestinal cells

**Figure 7.**
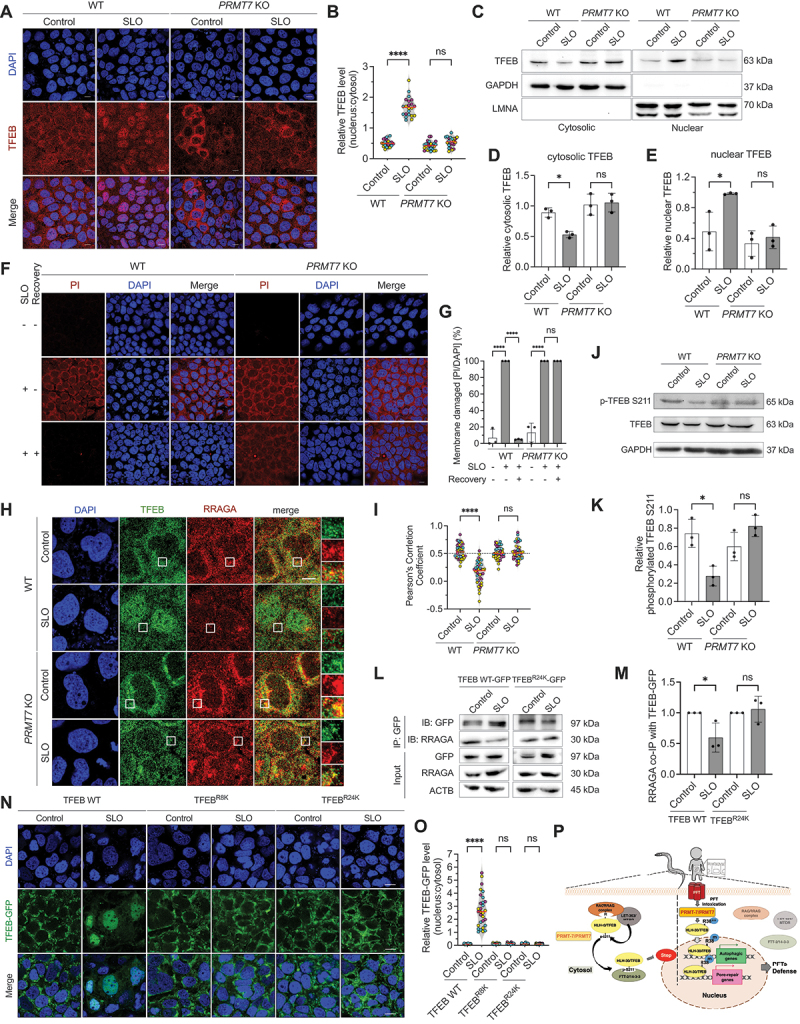
PRMT7 activates INCED in response to SLO intoxication by promoting TFEB nuclear translocation in human cells. (A) the representative images of TFEB in WT and *PRMT7* KO cells treated with SLO or control for 25 min. DAPI indicates nucleus staining. Scale bars: 10 μm. (B) the quantification of the nuclear:cytoplasmic ratio of the TFEB intensity in WT and *PRMT7* KO cells treated with SLO or control for 25 min (*n* = 21 in WT-control, *n* = 22 in WT-SLO, *n* = 24 in *PRMT7* KO-control, *n* = 22 in *PRMT7* KO-SLO). (C) the representative WB images of cytosolic TFEB and GAPDH as well as nuclear TFEB and LMNA (lamin A/C) in WT and *PRMT7* KO cells treated with SLO or control for 25 min. (D) the quantification of cytosolic TFEB expression in WT and *PRMT7* KO cells treated with SLO or control for 25 min (*N* = 3). The fold change of cytosolic TFEB is normalized with GAPDH. (E) the quantification of nuclear TFEB expression in WT and *PRMT7* KO cells treated with SLO or control for 25 min (*N* = 3). The fold change of nuclear TFEB is normalized with LMNA. (F) the representative images of the pore-repair assay. The red signal indicates the distribution of propidium iodine (PI) in the WT and *PRMT7* KO cells treated with SLO or control for 25 min. DAPI indicates nucleus staining. Scale bar: 10 μm. (G) the percentage of membrane damaged cells in WT and *PRMT7* KO cells treated with SLO or control (*n* = 158, *N* = 3 in WT-control, *n* = 113, *N* = 3 in WT-SLO, *n* = 165, *N* = 3 in WT-SLO with recovery, *n* = 118, *N* = 3 in *PRMT7* KO-control, *n* = 142, *N* = 3 in *PRMT7* KO-SLO, *n* = 110, *N* = 3 in *PRMT7* KO-SLO with recovery). (H) the representative confocal images of TFEB and RRAGA in WT and *PRMT7* KO cells treated with SLO or control for 25 min. The enlarged images (right panels) are from the frame on the TFEB, RRAGA and merge images. DAPI indicates nucleus staining. Scale bar: 10 μm. (I) the Pearson’s correlation coefficient analysis of TFEB and RRAGA from WT and *PRMT7* KO cells treated with SLO or control for 25 min (*n* = 40 in WT-control, *n* = 40 in WT-SLO, *n* = 40 in *PRMT7* KO-control, *n* = 40 in *PRMT7* KO-SLO). Pearson’s correlation coefficient: −1 to 0 indicates negative correlation, 0 to 0.5 indicates no correlation, and > 0.5 indicates positive correlation. (J) the representative WB images of phosphorylated TFEB S211, TFEB and GAPDH in WT and *PRMT7* KO cells treated with SLO or control for 25 min. (K) the quantification of phosphorylated TFEB S211 expression in WT and *PRMT7* KO cells treated with SLO or control for 25 min (*N* = 3). The fold change of phosphorylated TFEB S211 is normalized with TFEB. (L) the representative WB images of co-immunoprecipitation of TFEB-GFP and RRAGA in TFEB WT and TFEB^R24K^ cell. (M) the quantification of RRAGA pulled-down with TFEB-GFP in TFEB WT and TFEB^R24K^ cell. The fold change of co-IP RRAGA is normalized with immunoprecipitated TFEB-GFP. (N) the representative images of TFEB-GFP in TFEB WT, TFEB^R8K^ and TFEB^R24K^ cells treated with SLO or control for 25 min. DAPI indicates nucleus staining. Scale bars: 10 μm. (O) the quantification of the nuclear:cytoplasmic ratio of the TFEB-GFP intensity in TFEB WT, TFEB^R8K^ and TFEB^R24K^ cells treated with SLO or control for 25 min (*n* = 30 in every experiment group). (P) a schematic diagram illustrating a plausible activation mechanism of the HLH-30/TFEB-dependent intrinsic cellular defense (INCED) by the posttranslational arginine (R) methylation on HLH-30/TFEB induced by PRMT-7/PRMT7. Data information: all data statistics except fig. 7 M based on: *p < 0.05 and ****p < 0.0001 by two-way ANOVA. Fig. 7 M statistic based on: *p < 0.05 by unpaired *t* test (two-tailed). ns represents non-significance. Means are shown in red lines. Each data set of an independent biological repeat was represented by a different color. See also fig. S7. Source data are available online for this figure.

Finally, we aimed to examine whether the above findings in *C. elegans* are evolutionarily conserved in humans. To this end, we used human Caco-2 intestinal epithelial cells as the *in vitro* cell model and created a Caco-2 *PRMT7* KO cell line by the CRISPR-Cas9 method (Fig. S7A and S7B). Our immunocytochemistry (ICC) staining images demonstrated that the PFTs, including streptolysin O (SLO) from *Streptococcus pyogenes* and Ahh1 from *Aeromonas dhakensis*, both robustly induced TFEB nuclear localization, while the *PRMT7* knockout significantly abolished the PFT-induced TFEB nuclear localization (Figures. 7A–B and S7C-S7D). To further reconfirm our ICC results, we performed nuclear and cytosolic protein fractionation and monitored the cellular distribution of TFEB in Caco-2 WT and *PRMT7* KO cells treated with SLO by Western blotting. Our results reconfirmed that SLO significantly induced the redistribution of TFEB from the cytosol into the nucleus in Caco-2 WT cells, while the SLO-induced TFEB nuclear localization was significantly abolished in the *PRMT7* knockout cells (Figure 7C, and quantification in 7D and 7E). Together, our results demonstrated that PRMT7 is required for TFEB nuclear translocation in response to PFTs intoxication in human intestinal cells.

To reconfirm the function of PRMT7 in INCED against PFTs in human intestinal cells, we first treated wild-type and *PRMT7* KO cells with either SLO or Ahh1 and monitored the pore-repair activity after recovery through propidium iodine (PI) staining. Our results displayed that, compared to wild-type cells, PI signal penetrated into the cytosol of *PRMT7* KO cells after recovery for 24 h, suggesting PRMT7 knockout compromised the ability of INCED against PFTs intoxication (Figures. 7F,G and S7E-S7F). Furthermore, to reconfirm the role of PRMT7 in the autophagy-dependent INCED in human intestinal cells, we monitored the autophagic markers SQSTM1/p62 and LC3B in the SLO treated cells (Fig. S7G-S7I). Our results showed that SLO activates autophagy in wild-type cells, as indicated by the increase in SQSTM1 degradation as well as LC3B-II upregulation. On the contrary, SQSTM1 degradation and LC3B-II formation were significantly inhibited in *PRMT7* KO cells after SLO intoxication, indicating a defect in autophagy induction. Moreover, our qRT-PCR analysis for the autophagy gene *MAP1LC3B/lgg-1* and the pore-repair gene *TRIO/unc-73* in wild-type and *PRMT7* KO cells treated with SLO demonstrated that PRMT7 is also required for the induction of autophagic and pore-repair genes in protecting PFT intoxication (Fig. S7J-S7K). Taken all together, our results suggested that PRMT7 also participates in activating INCED, such as autophagy and pore repair against PFT-caused plasma membrane damage, in human intestinal cells.

Next, to reconfirm the contribution of PRMT7 in the RRAGA-dependent regulation of TFEB cellular distribution, we first examined the potential colocalization of TFEB and RRAGA in Caco-2 wild-type and *PRMT7* KO cells upon SLO intoxication (Figure 7H,I). Our ICC results clearly indicated that SLO not only promotes TFEB nuclear localization but also significantly reduces the cellular colocalization of TFEB and RRAGA in the cytosol of wild-type cells (Pearson’s correlation coefficient < 0.5). However, the cytosolic colocalization between TFEB and RRAGA remained positive in *PRMT7* KO cells under SLO-treated conditions. Moreover, given that inhibition of the interaction between TFEB and RRAGA leads to the reduction of phosphorylation of serine 211 in TFEB by MTOR, we also monitored the phospho-TFEB S211 levels in these cells. Our results showed that while SLO intoxication can significantly reduce the p-TFEB S211 level in Caco-2 wild-type cells, the p-TFEB S211 level was not significantly diminished in *PRMT7* KO cells (Figure 7J,K). Taken all together, these data demonstrated that PRMT7 is also required for the dissociation between TFEB and RRAGA in response to SLO intoxication.

Next, we examined whether PRMT7-dependent arginine methylation is also required for the disruption of interaction between TFEB and RRAGA by PFT in human cells. Given that the arginine 24 in the RRAGA binding domain of TFEB is conserved to the arginine 38 in *C. elegans* (as shown in Fig S6A), we generated HeLa cells that expressed TFEB WT-GFP or the arginine methylation-deficient TFEB^R24K^-GFP. Our immunochemical (IP-WB) analysis demonstrated that while SLO treatment can significantly reduce the interaction between TFEB WT-GFP and RRAGA, the arginine methylation-deficient mutation of TFEB at arginine 24 can significantly reverse this phenomenon (Figure 7L,M). Interestingly, this arginine to lysine mutation also significantly abolished the induced interaction between TFEB and PRMT7 by SLO in Caco-2 cells (Fig. S7L-7 M), suggesting that arginine 24 of TFEB is not only a pivotal amino acid for controlling the interaction between TFEB and RRAGA but also a potential substrate for methylation by PRMT7. Moreover, the control experiments demonstrated the specific interactions between TFEB WT-GFP with endogenous RRAGA and PRMT7 were not due to effect of GFP-tagging (Fig. S7N).

Finally, to reconfirm the notion that arginine methylation is required for the SLO-PFT induced nuclear localization of TFEB, we monitored the cellular distribution of TFEB WT-GFP, TFEB^R24K^-GFP, and TFEB^R8K^-GFP in Caco-2 cells treated with SLO. Of note, arginine 8 of TFEB has not only been demonstrated to be methylated in human cells [25] but has also been suggested as a key arginine residue for the interaction with RRAG GTPase complex [19]. Our ICC results clearly indicated that while SLO treatment can significantly induce nuclear localization of TFEB WT-GFP, the arginine methylation-deficient mutations of TFEB at arginine 8 as well as arginine 24 can significantly reverse this phenomenon (Figure 7N,O). Taken all together, our observations demonstrated that PRMT7 is not only a key regulator of HLH-30/TFEB through modulation of the RRAG GTPases-MTORC1 sequester system but also evolutionarily conserved in both *C. elegans* and humans to maintain host plasma membrane integrity compromised by bacterial PFTs.

## Discussion

Plasma membrane integrity is pivotal to cell function and survival. Therefore, cells targeted by PFTs need to mount instant and intricate cellular defenses against these detrimental assaults [1]. We previously unveiled that the PFT-elicited HLH-30/TFEB-dependent INCED systems are essential for damaged plasma membrane repair in *C. elegans* intestinal epithelium [5]. Here, we demonstrated that the activity of PRMT-7 is indispensable to the activation of this HLH-30/TFEB-dependent INCED. A model summarizing our findings is depicted in Figure 7P. HLH-30/TFEB, a master transcription factor for the organellar functions of lysosome and autophagy, is tightly regulated by the RRAG GTPases-MTORC1-YWHA/14-3-3 sequester system in the cytosol [19]. Here, we revealed that PRMT-7 participates in the posttranslational arginine methylation of HLH-30 on its RRAG complex binding domain to promote the nuclear localization of HLH-30 upon PFT intoxication in *C. elegans*. Moreover, we demonstrated that PRMT7 is also evolutionarily conserved to regulate TFEB nuclear localization and repair plasma damage caused by PFTs in human intestinal cells. Together, our study not only unveils a novel PRMT7-dependent post-translational regulation of HLH-30/TFEB but also demonstrates an evolutionarily conserved epithelium INCED against the most common mode of bacterial virulence factor, bacterial pore-forming toxin, in metazoans.

Important transcription factors, such as TP53/p53, are subjected to multiple levels of regulation, including many different types of post-translational regulations (PTRs), to properly fine-tune their activities in response to various cellular stimuli [36]. As an essential transcription factor for lysosomal and autophagic functions, HLH-30/TFEB participates in many normal physiological functions, including energy homeostasis, stress and immune responses, and development [17]. Therefore, TFEB dysregulation has been linked to a variety of human diseases, including lysosomal storage disorders, metabolic disorders, neurodegenerative diseases, and cancers. Herein, we identified specifically a novel PTR of HLH-30 mediated by PRMT-7 in response to bacterial PFT intoxication through the methylation of its arginine 38 in the RRAGA binding domain. Given that HLH-30/TFEB is a central regulator for cellular homeostasis, its dynamics and function are exquisitely regulated in response to different environmental cues [17]. Thus, it will be of great interest to know whether this specific PTM is also required for the regulation of HLH-30/TFEB in response to stimuli, other than PFTs, in the above-mentioned physiological processes and pathological conditions. Moreover, it has not escaped our notice that protein nuclear localization can also be regulated by posttranslational arginine methylation through the regulation of its liquid-liquid phase separation (LLPS) and interaction with the nuclear pore complex (NPC) [37]. Herein, we clearly described the role of arginine 38 methylation in HLH-30 in antagonizing the cytosolic RRAG GTPases-MTORC1-YWHA/14-3-3 sequester system for its nuclear localization, but we did not rule out the possibility that this specific PTM may also participate in LLPS or direct interaction of HLH-30 with nuclear transport regulators, as suggested by our results in Figure 4B,C. Since LLPS of TFEB has been reported [15], further investigations will be needed to corroborate this notion.

The dynamics and function of protein arginine methylation are mediated by its writers (PRMTs), erasers (arginine demethylases), and readers (proteins with the Tudor, WD40, or PHD domains that specifically recognize the methylated arginine motifs) [26,27]. In this study, we demonstrated, to our knowledge, for the first time that PRMT-7 participates in the post-translational arginine methylation of HLH-30. Thus, it is warranted for future research to identify the erasers (arginine demethylases) that control the dynamics of this specific PTM on HLH-30/TFEB and the readers that may also interpret and relay this molecular signal. Moreover, arginine methylation is not only an important epigenetic mark on histone proteins but also a functional PTM on various transcription factors that contributes to the precision regulation of transcriptome. Epigenetic regulators and transcriptional coregulators with Tudor, WD40, or PHD domains that bind specific methyl-arginine motifs can serve as functional readers to interpret these specific PTMs and regulate transcription activity [26]. Intriguingly, we also identified the PRMT-7-dependent arginine 174 methylation of HLH-30 that is not mediated in the regulation of HLH-30 nuclear localization. Thus, it will be of particular interest to examine whether this specific PTM may participate in the transcriptional activity of HLH-30 in the nucleus in response to bacterial PFT intoxication.

In summary, we discovered that the PRMT7-HLH-30/TFEB signal axis regulates epithelial INCED against bacterial PFT *in vivo* and in human cells. Our study demonstrated that how studying PFT pathogenesis in *C. elegans* can reveal astonishing intricacy and novel connections among intracellular signaling pathways. Given that abnormal regulations of PRMT7 and TFEB have been linked to a variety of human diseases, our findings about the PRMT7-TFEB signal transduction may not only advance our knowledge about INCED against PFT intoxication at the organismal level but also shed light on the potential molecular basis of these human diseases.

## Materials and methods

### Elegans and bacterial strains

The *C. elegans* strains, bacterial strains, and plasmids used in this study are listed in Tables S1, S2 and S3, respectively. The *C. elegans* strains used in this study were generated by the standard genetic method and maintained on nematode growth (NG) plates using *E. coli* strain OP50 as the normal food source under standard conditions [38]. The *E. coli* strains expressing Cry5B, Cry21A, and Ahh1 were used in Cry-PTFs toxicity experiments, and all bacterial strains were cultured under standard conditions [39].

### Generation of the *C. elegans* strains

YQ348 *prmt-1(tm3613);wgls433[hlh-30:TY1:EGFP:3×FLAG+unc-119(+)]*, YQ339 *prmt-4(tm4959);wgls433[hlh-30:TY1:EGFP:3×FLAG+unc-119(+)]*, YQ352 *prmt-5(tm6220);wgls433[hlh-30:TY1:EGFP:3×FLAG+unc-119(+)]*, YQ351 *prmt-6(tm5240);wgls433[hlh-30:TY1:EGFP:3×FLAG+unc-119(+)]*, YQ341 *prmt-7(tm4890);wgls433[hlh-30:TY1:EGFP:3×FLAG+unc-119(+)]*, YQ353 *prmt-9(tm6293);wgls433[hlh-30:TY1:EGFP:3×FLAG+unc-119(+)]*, YQ349 *prmt-7(tm4890);adls2122[lgg-1*_*p*_*::lgg-1*-GFP *+ rol-6(su1006)]*, YQ534 *raga-1(ok386);unc-119(tm4063);wgls433[hlh-30:TY1:EGFP:3×FLAG+unc-119(+)]*, and YQ592 *ragc-1(tm1974); unc-119(tm4063);wgls433[hlh-30:TY1:EGFP:3×FLAG+unc-119(+)]* were generated by crossing the *wgls433* transgene into each mutation background by the standard genetic method [40]. YQ621 *prmt-7(tm4890);sqIs19 [hlh-30p:hlh-30:GFP + rol-6(su1006)]* were generated by crossing the *sqIs19* transgene into *prmt-7(tm4890)* background. YQ432 *unc-119(tm4063);wfEx351[prmt-7*_*p*_*::dGFP:unc-54+unc119(+)]* was created by microinjection of the *prmt-7*_*p*_*::dGFP:unc-54+unc119(+)* transgene from the *pwf351* plasmid into the *unc-119(tm4063)* background. YQ482 *prmt-7(tm4890);unc-119(tm4063);wgls433[hlh-30:TY1:EGFP:3×FLAG+unc-119(+)];wfEx409[app-1*_*p*_*::prmt-7*^*D143A,E145A*^*::unc-54];myo-2*_*p*_*::mCherry:myo-2*, YQ483 *prmt-7(tm4890);unc-119(tm4063);wgls433[hlh-30:TY1:EGFP:3×FLAG+unc-119(+)];wfEx409[app-1*_*p*_*::prmt-7 WT:unc-54];myo-2*_*p*_*::mCherry:myo-2*, YQ454 *unc-119(tm4063);wgls433[hlh-30:TY1:EGFP:3×FLAG+unc-119(+)];wfEx442[app-1*_*p*_*::mCherry:prmt-7];myo-2*_*p*_*::mCherry:myo-2*, and YQ565 *unc-119(tm4063);wgls433[hlh-30:TY1:EGFP:3×FLAG+unc-119(+)];wfEx473[app1*_*p*_*::raga-1:mCherry:raga-1];myo-2*_*p*_*::mCherry:myo-2* were generated by microinjection of the *app-1*_*p*_*::prmt-7*^*D143A,E145A*^*::unc-54*, *app-1*_*p*_*::prmt-7 WT:unc-54*, *app-1*_*p*_*::mCherry:prmt-7* and *app1*_*p*_*::raga-1:mCherry:raga-1* transgenes respectively from the *pwf409, pwf503, pwf442*, and *pwf473* plasmid into the *hlh-30:TY1:EGFP:3×FLAG* background. YQ479 *hlh-30(tm4890);unc-119(tm4063);wfEx407[app-1*_*p*_*::hlh-30 WT:mCherry:unc-54+unc-119(+)];myo-2*_*p*_*::GFP:myo-2*, YQ554 *hlh-30(tm1978);unc-119(tm4063);wfEx467[app-1*_*p*_*::hlh-30*^*R38K*^*::mCherry:unc-54+unc-119(+)];myo-2*_*p*_*::GFP:myo-2*, and YQ600 *hlh-30(tm1978);unc-119(tm4063);wfEx504[app-1*_*p*_*::hlh-30*^*R174K*^*::mCherry:unc-54+unc-119(+)];myo-2*_*p*_*::GFP:myo-2* were made by microinjection of the *app-1*_*p*_*::hlh-30 WT:mCherry:unc-54*, *app-1*_*p*_*::hlh-30*^*R38K*^*::mCherry:unc-54* and *app-1*_*p*_*::hlh-30*^*R174K*^*::mCherry:unc-54* transgenes respectively from the *pwf407, pwf467*, and *pwf504* plasmid into the *hlh-30(tm4890)* background. YQ596 *hlh-30(tm1978);unc-119(tm4063);wfEx467[app-1*_*p*_*::hlh-30*^*R38K*^*::mCherry:unc-54+unc-119(+)];myo-2*_*p*_*::GFP:myo-2;wfEx493[vha-6*_*p*_*::raga-1:EGFP:raga-1+rol-6(su1006)]* were created by microinjection of the *vha-6*_*p*_*::raga-1:EGFP:raga-1* transgene from the *pwf493* plasmid into the *app-1*_*p*_*::hlh-30*^*R38K*^*::mCherry:unc-54* background. YQ601 *hlh-30(tm1978);unc-119(tm4063);wfEx407[app-1*_*p*_*::hlh-30 WT:mCherry:unc-54+unc-119(+)];myo-2*_*p*_*::GFP:myo-2;wfEx493[vha-6*_*p*_*::raga-1:EGFP:raga-1+rol-6(su1006)]* were generated by microinjection of the *vha-6*_*p*_*::raga-1:EGFP:raga-1* transgene from the *pwf493* plasmid into the *app-1*_*p*_*::hlh-30 WT:mCherry:unc-54* background. The plasmids used in the generation of these *C. elegans* strains were listed in the Appendix Table S3.

### PFT intoxication in *C. elegans*

#### PFT plate preparation

The *E. coli* strains OP50 or XL-10-Gold carrying the pQE9, pQE9-Cry5B, pQE9-Cry21A, and pET30a-Ahh1 plasmids were cultured in LB broth with 5 μg/mL carbenicillin (Sigma-Aldrich 4,800,946) for 17 h at 37°C. After overnight culture, the broth was diluted with LB at 1:4 ratio and incubated at 37°C for 1 h for refreshment. Then, the *E. coli* OP50 broth was incubated with 50 μM isopropyl-β-D-1-thiogalactopyranoside (IPTG; Cyrusbioscience, 101-367-93-1) at 30°C for 3 h (4.5 h for *E. coli* strain XL-10-Gold) to induce PFT expression. After induction, 30 μL of bacterial broth was spread on each NG-IC plate (NG plates with 50 μg/mL carbenicillin and 50 μM IPTG) and cultured overnight at 25°C. Next day, these PFT plates were ready for all PFT experiments for *C. elegans*.

#### PFT intoxication

Synchronized late L4-young adult stage *C. elegans* animals were fed either on control plates with *E. coli* that did not express PFT (vector control) or on plates with *E. coli* expressing PFT (Cry5B, Cry21A, or Ahh1) at 25°C for the indicated time in each experiment.

#### Quantitative cry-PFTs susceptibility

Synchronized late L4-young adult stage *C. elegans* animals were fed with *E. coli* XL-10-Gold that did not express Cry-PFT (pQE9 vector control) or *E. coli* XL-10-Gold expressing Cry-PFT (pQE9-Cry5B or Cry21A) and incubated at 25°C. The mortality of worms on each plate was scored every 24 h. Animals were scored at the indicated times and considered dead upon failure to respond to touch. Animals missing from the agar plate were censored on the day of loss. Animal survival was normalized and plotted as a nonlinear regression curve by Prism 9.0 (GraphPad Software).

#### Plasma membrane pore-repair assay

Synchronized late L4-young adult stage animals were fed with *E. coli* OP50 that did not express Cry-PFT (pQE9 control) or *E. coli* OP50 expressing Cry-PFT (pQE9-Cry5B or Cry21A) for 30 min at 25°C and then transferred to normal *E. coli* OP50 (non-toxin) plates for 30 min or 24 h for recovery. After recovery, the worms were soaked in 1 μM SYTOX blue (Invitrogen, S34857) or 6.7 μg/mL propidium iodine (PI; Sigma-Aldrich 25,535,164) mixed with 5 mg/mL serotonin (Sigma-Aldrich, 61-47-2) solution for 45 min at 20°C, followed by washing twice with M9 buffer. SYTOX blue or PI signals in these animals were acquired by a Nikon Eclipse Ti inverted microscope system.

### RNA interference (RNAi)

*coli* strain HT115 transformed with RNAi plasmids were spread on NG-IC plates and incubated at 25°C overnight to induce the dsRNA expression. *E. coli* HT115 with L4440, an empty vector, was used as a negative control for RNAi. Synchronized *C. elegans* L1 larvae were cultured on *E. coli* plates containing L4440 or RNAi clones expressing dsRNA of the gene of interest at 20°C until the L4-young adult stage. These L4-young adult stage worms were then transferred to NG-IC plates with Cry-PFTs bacteria mixed with RNAi bacteria at a 3:1 ratio (30 μL per plate) at 25°C for the indicated time in each experiment. The *C. elegans* RNAi feeding clones were from the Ahringer RNAi library, and all RNAi clones have been confirmed by plasmid DNA sequencing.

### *C*.elegans fluorescent imaging

#### Cellular distribution of HLH-30

Synchronized late L4-young adult stage *C. elegans* animals expressing the *hlh-30:GFP* or *hlh-30:mCherry* transgene were fed with *E. coli* OP50 that did not express PFT (vector control) or *E. coli* OP50 expressing PFT (Cry5B, Cry21A, or Ahh1) for 1 h at 25°C to induce HLH-30 nuclear localization. HLH-30:GFP or HLH-30:mCherry signals in these animals were acquired by the Nikon Eclipse Ti inverted microscope system, and animals with more than 10 intestinal cells that demonstrated nuclear HLH-30 were defined as positive for HLH-30 nuclear localization. Cytosolic and nuclear HLH-30:GFP intensity in these animals was quantified using ImageJ (National Institutes of Health). In brief, the areas of interest were enclosed by rectangles and added into the ROI manager of the software. The GFP intensity mean value of ROIs was analyzed by the Measurement tool of ImageJ.

#### In vivo *transcriptional reporter analysis*

Synchronized late L4-young adult stage YQ432 animals carrying the *prmt-7*_*p*_*::dGFP* transgene were fed with *E. coli* OP50 that did not express Cry-PFT (pQE9 control) or *E. coli* OP50 expressing Cry-PFT (pQE9-Cry5B) for 3 h at 25°C and the GFP signals were acquired by the Nikon Eclipse Ti inverted microscope system. GFP intensity was quantified by Image-Pro Plus 7.0 (Media Cybernectics). In brief, gray scale images were used, and the dGFP-expressing areas were defined through the Intensity Range Selection (Threshold value: 400–4095) and calculated by the Count and measure object tool of the software.

#### Autophagy analysis

All autophagy analyses were performed according to the established guidelines [32,41]. GFP:LGG-1 quantification: Synchronized L4-young adult stage DA2123 and YQ349 animals carrying the *GFP:lgg-1* and *prmt-7(tm4890);GFP:lgg-1* transgenes respectively were fed with *E. coli* OP50 that did not express Cry-PFT (vector control) or *E. coli* OP50 expressing Cry-PFT (Cry5B or Cry21A) for 3 h at 25°C and GFP:LGG-1 signals were acquired by an Olympus confocal laser scanning microscope (FV3000) system. GFP intensity was quantified using Image-Pro Plus 7.0. In brief, gray scale images were used, and the GFP:LGG-1 expressing areas were defined through the Intensity Range Selection (Threshold value: 500–4095 for DA2123 and 600–4095 for YQ349) and calculated by the Count and measure object tool of the software. GFP:LGG-1 foci number in the Int1 (the first anterior ring of the intestine) cells was determined by ImageJ. In brief, 8-bit images were used and the GFP:LGG-1 puncta areas should be segmented against the background by a threshold value. The chosen area number was counted by the Analyze particles tool of the software. SQST-1:GFP quantification: Synchronized L4-young adult stage HZ946 animals carrying *sqst-1:GFP* were first knocked down *prmt-7* by RNAi and then fed with *E. coli* OP50 that did not express Cry-PFT (vector control) or *E. coli* OP50 expressing Cry-PFT (Cry5B or Cry21A) for 3 h at 25°C. SQST-1:GFP signals were acquired by the Olympus FV3000 confocal laser scanning microscope system. The SQST-1:GFP foci number in the Int1 (the first anterior ring of the intestine) cells was determined by ImageJ as described in the GFP:LGG-1 quantification.

### Colocalization analysis

Synchronized L4-young adult stage YQ454 animals carrying *hlh-30:GFP;mCherry:prmt-7* were fed with control or Cry5B for 5, 10, and 15 min, followed by images captured by the Olympus FV3000 confocal laser scanning microscope system. In the EV or *prmt-7* RNAi knockdown L4-young adult stage YQ565 animals carrying *hlh-30:GFP;raga-1:mCherry*, the YQ596 animals carrying *hlh-30*^*R38K*^*::mCherry;raga-1:GFP*, and the YQ601 animals carrying *hlh-30 WT:mCherry;raga-1:GFP*, these worms were fed with control or Cry5B for 1 h and photographed. The quantification of GFP and mCherry signal colocalization was analyzed by ImageJ. The Image-Color-Split Channels menu command was used to separate the z stack for the two fluorescence channels of the confocal microscope image. Then, channel colors were assigned by the Image-Color-Channels Tool menu command. Pearson’s correlation coefficient value for the two split channels was calculated using Plugins-JACoP. The interpretations for the results of Pearson’s correlation coefficient were that + 1 for perfect correlation, 0 for no correlation, and − 1 for perfect anti-correlation [42].

### Förster/Fluorescence resonance energy transfer (FRET) analysis

Synchronized late L4-young adult stage YQ454 animals carrying *hlh-30:EGFP;mCherry:prmt-7* were fed with control or Cry5B for 30 min, followed by the FRET image captured. In the EV or *prmt-7* RNAi knockdown L4-young adult stage YQ565 animals carrying *hlh-30:EGFP;raga-1:mCherry*, YQ596 animals carrying *hlh-30*^*R38K*^*::mCherry;raga-1:EGFP*, and L4 YQ601 animals carrying *hlh-30 WT:mCherry;raga-1:EGFP*, these worms were fed with control or Cry5B for 1 h and photographed. FRET was performed on the Olympus confocal laser scanning microscope (FV3000) system. In brief, a reference picture was first captured within an adequate parameter. The excitation laser for acceptor protein fluorescence was turned off, and all the sample images were acquired with the same parameters as the reference image. The FRET images were analyzed by cellSens (Olympus). The donor sample and accepter sample were input into the FRET correlation tool and analyzed areas against background in both reference and sample images were segmented by the ROI signal and ROI background tool of the software. After that, the FRET analysis tool was used to quantify the FRET signal through the Gordon (1998) computation method [43–46]. The FRET image resulting from the Gordon method is a gray-value image with automatic pseudocolor. High values are displayed in red, while low ratio values are displayed in magenta.

### Fluorescence recovery after photobleaching (FRAP) analysis

Synchronized L4-young adult stage OP433, YQ340, YQ483, and YQ482 animals carrying the *hlh-30:GFP* transgene were fed with Cry5B or control for 30 min. After Cry5B treatment, these animals were immobilized with 1 mM levamisole (MerckMillipore 359,302.1566) and fixed on 1% agarose gel pads. Photobleaching and image capture were performed on the Olympus confocal laser scanning microscope (FV1000) system using the 100X objective. The 405-nm laser was used to bleach a selective region of the HLH-30:GFP signal, with HLH-30:GFP fluorescence detected by the 488-nm excitation laser.

### Quantitative real-time PCR (qRT-PCR)

#### Worm

Synchronized L4-young adult stage N2 wild-type or *prmt-7(tm4890)* animals were fed with *E. coli* OP50 that did not express Cry-PFT (vector control) or *E. coli* OP50 expressing Cry-PFT (pQE9-Cry5B) for 3 h at 25°C to induce the expression of *prmt-7*, the HLH-30-dependent autophagic genes (*lgg-1* and *atg-18*), and the HLH-30-dependent membrane repair genes (*ced-1* and *unc-73*). Total RNA was extracted using 500 μL TRIzol (Invitrogen 15,596,018) from 3000 worms and reverse transcribed by M-MLV reverse transcriptase (Promega, M1708) using random hexamer primers. The complementary DNA (cDNA) was then subjected to qRT-PCR analysis using SYBR green detection (Roche 4,913,914,001) on a 7500 Fast Real-Time PCR System (Applied Biosystems). The experiment was performed using three independent sets of cDNA. Fold change was calculated using the Pfaffl method [47], and the relative expression between samples was normalized to *eft-2* as the reference gene [48].

#### Cell

2.5 × 10^5^ *PRMT7* WT or *PRMT7* KO cells were seeded into a 6-well cell culture dish. Until 70–80% confluency, cells were treated with 0.8 μg/mL SLO for 50 min. Total RAN was extracted using 400 μL TRIzol for each well and reverse transcribed by M-MLV reverse transcriptase using random hexamer primers. cDNA detection was described in worm section. The relative expression between samples was normalized to *GAPDH* as the reference gene. The qRT-PCR primers used in this assay are listed in Table S4.

### Mass spectrometry (MS) analysis

#### Protein collection

Synchronized late L4-young adult stage OP433 animals carrying the *hlh-30:GFP* transgene were fed with control or Cry5B for 1 h. About 20,000 worms were collected and frozen with homogenizer buffer (HB buffer; 20 mM HEPES, pH 7.9, 100 mM NaCl, 10 mM KCl, 0.1 mM EDTA, 1.5 mM MgCl_2_, 0.5 mM EGTA, 44 mM sucrose, 0.5% octyl phenol ethoxylate [JT Baker_®_, X198–07], and protease inhibitor tablet [Roche 04,693,159,001]) at − 80°C overnight. Frozen pellets were thawed with 3–5 times the volume of HB buffer and homogenized by tissue grinder (KIMBLE_®_ KONTES_®_ Dounce Tissue Grinder 885,300–0007). Following centrifugation at 18,800 × g for 15 min, supernatants were collected and immunoprecipitated with GFP-Trap_®_ Magnetic Agarose (Chromotek, gtma) at 4°C overnight. After immunoprecipitation, magnetic agarose beads were resuspended by 20 μL 1 × SDS-sample buffer (2% SDS, 10% glycerol, 50 mM β-mercaptoethanol, 0.005% bromophenol blue, and 0.05 M Tris HCl, pH 6.8) and boiled for 10 min to dissociate HLH-30:GFP from GFP-Trap beads. Immunoprecipitated HLH-30:GFP proteins were applied to SDS-PAGE electrophoresis. After electrophoresis, the gel was stained using Coomassie blue for 45 min and destained by the destaining solution (40% methanol, 10% acetic acid) to confirm the quantity and molecular mass of HLH-30:GFP which is 83 kDa. A gel piece ranging from 75 to 100 kDa was cleaved for in-gel digestion. For in-gel digestion, the gel piece was destained using the destain solution (25 mM ammonium bicarbonate mixed with 100% methanol at a ratio of 1:1) for 3 h and washed with H_2_O for 5 min. Followed the wash, 100% acetonitrile was added and removed immediately after 3 min, and then the gel was dried using a centrifugal concentrator for 10 min. 100 μg of trypsin at 0.01 μg/μL was added. The sample was incubated at 37°C for 16 h. After incubation, an adequate mixed solution with 50% acetonitrile and 5% formic acid was added into the trypsin-treated gel, and sonicated for 20 min. The mixture was centrifuged at 13,000 g for 3 min and the supernatant was transferred to a new tube. The pellet was saved, and added to an adequate mixed solution with 50% acetonitrile and 5% formic acid, and sonicated for 20 min. After centrifugation at 13,000 g for 3 min, the supernatant was transferred to the previously collected one. The total supernatant was dried by the centrifugal concentrator and was ready for mass spectrometry analysis.

#### Mass spectrometry

The liquid chromatography-quadrupole time-of-flight mass spectrometry was performed using a Waters nanoACQUITY UPLC coupled to a Bruker Impact HD™ UHR-QTOF by Center for Research Resources and Development of Kaohsiung Medical University, Taiwan. For short, the peptide mixtures underwent on-line desalting using a short trapped column (Waters, Symmetry C18, 5 μm, 180 μm x 20 mm 186,006,527). The subsequent separation was carried out using a nano-flow reversed-phase C18 column (Waters, BEH C18, 1.7 μm, 75 μm x 250 mm 186,003,545). To initiate the process, a 3-min desalting step was performed at a flow rate of 10 μL/min with 0.1% (v:v) formic acid. Following this, the switching valve was automatically adjusted to the analytical position. Tryptic peptides were then separated using a long analytical column with a flow rate of 200 nL/min. The mobile phases consisted of 0.1% (v:v) formic acid for mobile phase A and 99.9% acetonitrile with 0.1% (v/v) formic acid for mobile phase B. The gradient conditions were as follows: from 0 to 2 min, the percentage of B increased from 0.5% to 5%; from 2 to 60 min, B increased from 5% to 45%; from 60 to 65 min, B decreased from 45% to 95%; from 65 to 70 min, B was held at 95%; from 70 to 71 min, B decreased from 95% to 0.5%; and from 71 to 90 min, B was held at 0.5%.The analytical column was connected to an electrospray ionization (ESI)-QTOF instrument (Bruker, Impact HD^TM^) equipped with an additional supply of acetonitrile vapor in nitrogen as an ionization enhancer (Bruker, nanoBooster^TM^). The mass spectrometer operated in the data-dependent acquisition mode, switching between full scan MS and MS/MS acquisition automatically. The following tune parameters were applied: Funnel 1 RF = 400Vpp, Funnel 2 RF = 600Vpp, Hexapole RF = 200Vpp, and pre-pulse = 10 µs. Collision energy was set at 7 eV, with a stepping ramp from 100% to 120% exclusively for MS/MS, aiding the fragmentation of larger endogenous peptides. Nitrogen (N_2_) was employed as the collision gas. The number of precursor ions was dynamically adjusted to fit within a fixed cycle time of 3 s to maintain a constant sampling rate across the chromatographic peak.

#### Data analysis

The data were analyzed using Compass DataAnalysis (Version 4.1 SR1) software which generated peak lists in mgf format. To identify proteins, tandem mass spectrometry peak lists were processed using Mascot software Version 2.5 from Matrix Science. The peptide sequences were matched against the *Caenorhabditis elegans* (*C. elegans*) taxonomy, consisting of 29,644 sequences obtained from the NCBI as of 20 December 2020. Mass tolerances for peptides and product ions were set to 50 ppm and 0.5 Da, respectively, with the instrument specified as “ESI-QUAD-TOF.” The protease designated was semi-trypsin, allowing for two missing cleavages. Carboxymethylation on cysteine was considered a fixed modification, while methionine oxidation was treated as a variable modification. Proteins were accepted if they had at least one Rank 1 peptide with a Mascot ion score of over 40.0 (*p* < 0.05).

### Site-directed mutagenesis

5 ng of plasmid was amplified by using PfuUltra High-Fidelity DNA Polymerase (Agilnet 600,380), 0.4 mM primers, pfu buffer, 0.25 mM dNTP and 6% dimethyl sulfoxide with the following program. Step1. 92°C for 2 min. Step 2. 92°C for 10 s → annealing temperature for 30 s → 68°C for 2 min/1 kb for 10 cycles. Step 3. 92°C for 10 s → annealing temperature for 30 s → 68°C for 2 min/1 kb (extending 10 s/cycle) for 20 cycles. After amplification, the product was digested by DpnI and transformed into *E. coli*competent cells. The correct mutant plasmids were then confirmed by plasmid DNA sequencing. The primer sets used in the generation of the *prmt-7*, *hlh-30*, and *TFEB* mutant transgenes are listed in Table S4.

### Molecular modeling

The models of CeRAGA-1 and CeRAGC-1 were constructed by using homology modeling with SWISS-MODEL [49]. The HLH-30 model, spanning residues M1 to P50, was generated through a combination of template-based SWISS-MODEL [49] and TopModel [50] approaches, with the RPTOR-TFEB-RRAG-Regulator complex (PDB: 7UX23) [51] as the template. To further investigate the potential interactions and conformations, these models were docked onto the structure of the RPTOR-TFEB-RRAG-Regulator complex (PDB: 7UX23) using the align command in PyMOL (Ver. 2.3.2) to create a predicted *C. elegans* HLH-30-RAGA-1-RAGC-1 complex model.

### Cell culture

Caco-2 cells (ATCC, CRL-2102) were cultured in Dulbecco’s modified Eagle’s medium (DMEM; Gibco™, 12100046) containing high glucose and L-glutamine supplemented with 10% fetal bovine serum (FBS; Gibco™, 10437028) and 10 μg/mL transferrin (Sigma-Aldrich 11,096-37-0) at 37°C in a humidified incubator with 5% CO_2_. 5 × 10^5^ cells were seeded into a 10 cm cell culture dish and routinely subcultured using 0.5% trypsin-EDTA (Genedirex, CC507–0100) when they reached to 70–80% confluence. Culture media were changed every other day. Monolayer cells became 70–80% confluent 4–6 days after the inoculum, and the cultures were stopped after 25 passages. For Caco-2 *PRMT7*^*KO*^ cells, 1 μg/mL puromycin (Sigma-Aldrich, 58-58-2) was added to high glucose DMEM containing 10% BSA and 10 μg/mL transferrin and maintained as described for the wild-type Caco-2 cells. HeLa cells (ATCC, CCL-2) were cultured in DMEM containing high glucose and L-glutamine supplemented with 10% FBS at 37°C in a humidified incubator with 5% CO_2_. 3 × 10^5^ cells were seeded into a 10 cm cell culture dish and routinely subcultured using 0.5% trypsin-EDTA when they reached to 70–80% confluence. Culture media were changed every other day. Monolayer cells became 70–80% confluent 2–3 days after the inoculum, and the cultures were stopped after 25 passages.

### Generation of the caco-2 *PRMT7* KO cell line

The EDCPV plasmid which contains an FKBP12-derived destabilizing domain to Cas9 (DD-Cas9) was acquired from Addgene (90085; deposited by Raffaella Sordella Lab) [52]. The filler sequence of EDCPV was removed for cloning with the desired sgRNA (5’-TGATGCTGCTGTGAAGATTG-3’) targeting the exon 6 of *PRMT7* and the Venus of EDCPV was replaced with a Puromycin resistance cassette. Caco-2 cells transfected with the EDCPV expressing *PRMT7* sgRNA were selected with 1 μg/mL puromycin (Sigma-Aldrich, 58-58-2). Shield 1 (CheminPharma, CIP-S1 -00,005) solubilized in 99% ethanol was then added at a concentration of 200 nM to stabilize DD-Cas9 to conduct CRISPR-mediated KO. The shield-1 treated transfected cells were collected and seeded into 10 cm dishes (2000 cells/dish) and single cell colonies were isolated and examined for the *PRMT7* KO cell line. The DNA sequence of the exon 6 of *PRMT7* in the *PRMT7* KO cell line was confirmed by Sanger sequencing and shown in Fig. S7B.

### Transient transfection of TFEB WT-GFP, TFEB^R8K^-GFP and TFEB^R24K^-GFP in HeLa cells

3 × 10^5^ HeLa cells were seeded into a 10 cm cell culture dish and transfected after 2 days (70 ~ 80% confluency). For transfection, 5 mL fresh DMEM with 10% FBS was added into cell culture dish 30 min prior to transfection. Fifteen μL PolyJet (SignaGen, SL100688) in 235 μL serum-free DMEM was added into 250 μL serum free DMEM containing 5 μg plasmid DNA and incubated in room temperature for 17 min. After incubation, the PolyJet and plasmid DNA mixture was added into the cell culture dish. After 5 h, the PolyJet/DNA complex-containing medium was replaced by DMEM with 10% FBS. The transfected cells were subcultured at the day post transfection for co-immunoprecipitation assay. All the plasmid used in this assay are listed in Appendix Tables S3.

### PFT intoxication in human epithelial cells

#### Streptolysin O (SLO) intoxication

Caco-2 and HeLa cells were incubated with DMEM containing 0.4 μg/mL SLO, prepared as described [53], and 5 mM dithiothreitol (DTT) (Sigma-Aldrich, 3 December 3483), for the reduction of disulfide bonds in streptolysin O (SLO), on ice for 30 min. Low temperatures promote SLO and cholesterol binding on the cell surface. After 30 min, the SLO-treated cells were then transferred to 32°C for 25 min for SLO oligomerization and perforation. For autophagy induction, cells were infected with SLO for 75 min.

#### Ahh1 intoxication

Recombinant hemolysin Ahh1, provided by Leadgene Biomedical Inc. (Case No. X22071201), was used to perforate Caco-2 cells. 1 μM Ahh1 was added into cell culture medium and incubated with cells at 37°C for 9 h for Ahh1 intoxication.

### Immunochemistry for protein analysis

#### Protein collection

5 × 10^5^ *PRMT7* WT or *PRMT7* KO cells were seeded into a 10 cm cell culture dish. Until 70–80% confluency (about 4–6 days), cells were infected with SLO as described in the streptolysin O intoxication section, and cell lysates were collected by scraping and followed by washing with PBS (Gibco™, 21600010) twice. Cell pellets were then incubated with 300 μL lysis buffer (1 × RIPA buffer (Millipore, 20–188) containing 1% protease inhibitor cocktail [Sigma-Aldrich, P3840] and 1% phosphatase inhibitor cocktail [Sigma-Aldrich, P0044]) on ice for 30 min and then sonicated for homogenization. After centrifugation at 18,800 × g for 15 min, supernatants were collected. Pierce™ BCA Protein Assay Kit (Thermo Scientific™, 23227) was used to determine protein concentration. 30 μg of total proteins mixed with 2 × SDS-sample buffer (4% SDS, 20% glycerol, 100 mM β-mercaptoethanol, 0.01% bromophenol blue and 0.1 M Tris HCl, pH 6.8) was boiled for 15 min and then ready for western blotting.

#### Nucleus and cytoplasm fractionation

Caco-2 cells were intoxicated with SLO and collected by scraping as described. The cell pellet was then gently resuspended and incubated with 200 μL cytosol buffer (1.5 μM MgCl_2_, 10 μM KCl, 10 μM pH 8.0 HEPES and 2 μM DTT) containing 2% protease inhibitor cocktail on ice for 10 min. The cell lysate was gently mixed by vortex for 30 s after 2 μL IGEPAL_®_ CA-630 (Sigma-Aldrich, I3021) was added and then centrifuged at 800 g for 8 min at 4°C. The supernatant was collected as cytosol fraction. The pellet as nucleus fraction was saved and washed with 1 mL ice-cold PBS and centrifuged at 800 g for 8 min at 4°C for 4 times. The washed pellet was incubated with 50 μL 1 × RIPA on ice for 10 min. After incubation, the pellet was sonicated for homogenization. Both cytosol and nucleus fraction were then centrifuged at 18,800 × g for 20 min at 4°C. The supernatants were collected for protein concentration determination as described and western blotting.

#### Co-immunoprecipitation

HeLa cells expressing TFEB-GFP were intoxicated with SLO and collected by scraping as described. The cell pellet was incubated with 500 μL IP lysis buffer (25 mM HEPES, pH 7.4, 120 mM NaCl, 5 mM EDTA and 1% octyl phenol ethoxylate) [19] on ice for 10 min and then sonicated for homogenization. The cell lysate was centrifuged at 18,800 × g for 20 min at 4°C. The supernatant was collected. 500 μg protein was immunoprecipitated with 10 μL GFP-Trap_®_ Magnetic Agarose at 4°C overnight. The immunoprecipitated TFEB-GFP was applied for western blotting detection.

#### Western blotting (WB)

Samples were separated by 12% SDS-PAGE (15% SDS-PAGE for LC3B). Then, proteins in polyacrylamide gels were transferred onto 0.45 μm PVDF membrane (Millipore, IEVH85R) (0.2 μm PVDF membrane (Millipore, ISEQ85R) for LC3B) using a semi-dry transfer system (Bio-Rad) at 300 mA for 45 min. After transferring, PVDF membranes were soaked with 5% nonfat milk for blocking for 1 h and then washed three times with Tris-Buffered Saline buffer (136.9 mM NaCl, 2.68 mM KCl, 24.76 mM Tris-Base, pH 7.4) containing 0.1% TWEEN_®_ 20 (MerckMillipore, 8.22184.0500; TBST). Primary antibodies were added and incubated with gentle shaking at 4°C overnight. After overnight incubation, PVDF membranes were washed three times with TBST and then secondary antibodies were added and incubated with gentle shaking at room temperature for 1 h. HRP substrates (Millipore, WBKLS0500) were used for visualization. All the antibodies used in this assay: monoclonal antibody to PRMT7 (Cell Signaling Technology 14,762), monoclonal antibody to LC3B (Cell Signaling Technology, 3868), polyclonal antibody to SQSTM1/p62 (FHbio, FH091384), monoclonal antibody to GFP (Roche 11,814,460,001), polyclonal antibody to phospho-TFEB (Ser211; Cell Signaling Technology 37,681), polyclonal antibody to ACTB/β-actin (GeneTex, GTX110564), monoclonal antibody to GAPDH (GeneTex, GT239), monoclonal antibody to RRAGA (Cell Signaling Technology, 4357), peroxidase AffiniPure Goat Anti-Rabbit IgG (H+L) (Jackson ImmunoResearch, 111-035-003) and peroxidase AffiniPure Goat Anti-Mouse IgG (H+L) (Jackson ImmunoResearch, 115-035-003).

### Immunocytochemistry (ICC)

Cells (1 × 10^5^) were seeded into a 12-well cell culture dish containing 18-mm round cover glass (MATSUNAMI, C018001). Until 70–80% confluency, cells were treated with SLO or Ahh1 as described in the SLO intoxication and Ahh1 intoxication section respectively. After SLO or Ahh1 intoxication, cells were fixed with 4% paraformaldehyde (Riedel-De Häen,16005) at room temperature for 20 min and washed three times using PBS. Then, cells were permeabilized with 0.4% octyl phenol ethoxylate for 15 min and washed three times using PBS. Next, cells were blocked with 1% bovine serum albumin (Sigma-Aldrich, A7906) in PBS for 1 h at room temperature. After blocking, cells were incubated with the primary antibody at 4°C overnight and then with the secondary antibody at room temperature for 1.5 h in the dark. Finally, cells were stained with DAPI (1:1000 dilution) in PBS for 10 min in the dark and mounted as described in the Plasma membrane pore-repair assay. Fluorescent images were acquired by the Olympus confocal laser scanning microscope (FV3000) system. The antibodies used in this assay: monoclonal antibody to RRAGA (Cell Signaling Technology, 4357), monoclonal antibody to TFEB (Cell Signaling Technology 91,767), goat Anti-Rabbit IgG H&L (Alexa Fluor® 594) (Abcam, ab150080) and goat Anti-Mouse IgG H&L (Alexa Fluor® 488) (Abcam, ab150113).

### Plasma membrane pore-repair assay in caco-2 cells

*PRMT7* WT or *PRMT7* KO Caco-2 cells (1 × 10^5^) were seeded onto a 12-well cell culture dish containing 18-mm round cover glass (MATSUNAMI, C018001). Until 70–80% confluency, cells were intoxicated by SLO or Ahh1, as described. After intoxication, cells were washed three times using DMEM. Cells were incubated with 50 μg/mL PI for 10 min and washed three times with PBS. 4% paraformaldehyde was used to fix the cells at room temperature for 20 min and then washed three times using PBS. Then, cells were stained with DAPI (1:1000 dilution) in PBS in the dark for 10 min. The fluorescence mounting medium (Dako, S3023) was used to mount the cells on a glass slide. For pore repair, the intoxicated cells were washed three times with PBS and incubated without SLO or Ahh1 for 24 h for recovery, followed by the PI staining and cells were fixed by the same procedure as described. Fluorescent images were acquired by the Olympus confocal laser scanning microscope (FV3000) system.

### Data analysis

All experiments were performed at least three times independently. For quantitative data, the *N* of independent experiments performed and the *n* of samples (e.g., animals or ROIs) examined per *N* are provided in the figure legend. Data are presented as mean ± SEM. Statistical analysis between two values was compared with an unpaired *t* test (two-tailed), and for 3 or more values with more than 1 independent variables was analyzed by two-way ANOVA. Survival analysis was performed using Prism 9.0 (GraphPad Software), and the Mantel – Cox log-rank test was used to assess the statistical significance of the difference in survival. Statistically significant differences of *p* < 0.05, *p* < 0.01, and *p* < 0.001 are represented by *, **, and ***, respectively.

## Abbreviations and acronyms

*A. dhakensis*: *Aeromonas dhakensis*
*atg/*ATG: autophagy-related (yeast Atg homolog)
bHLH-Zip: basic helix-loop-helix leucine zipper
*B. thuringiensis* (Bt): *Bacillus thuringiensis*
*C. elegans*: *Caenorhabditis elegans*
*ced*/CED: cell death abnormality
CePRMT-7: *Caenorhabditis elegans* PRMT-7
Cry toxins: crystal toxins
dGFP: degradable green fluorescent protein
EGFP: enhanced green fluorescent protein
FRAP: fluorescence recovery after photobleaching
FRET: fluorescence resonance energy transfer
GFP: green fluorescent protein
*hlh*/HLH: helix loop helix
HsPRMT7: *Homo sapiens* PRMT7
ICC: immunocytochemistry
INCED: Intrinsic cellular defense
IPTG: isopropyl-β-D-1-thiogalactopyranoside
LC-MS/MS: liquid chromatograph-tandem mass spectrometry
*lgg*/LGG: LC3 and GABARAP family
MiT family: microphthalmia family
MmPRMT7: *Mus musculus* PRMT7
MTORC1: mechanistic target of rapamycin kinase complex 1
NPC: nuclear pore complex
PFTs: pore-forming toxins
PI: propidium iodide
PMI: plasma membrane integrity
PRMTs: protein arginine methyltransferases
PTMs: post-translational modifications
RNAi: ribonucleic acid interference
SLO: streptolysin O
TFEB: transcription factor EB
qRT-PCR: quantitative real-time PCR
SQST-1/SQSTM1/p62: sequestosome 1
*unc*/UNC: uncoordinated

## Supplementary Material

PRMT7_supplement_122823 Autophagy R4.docx

## Data and materials availability

All materials generated in this study are available upon request. Source data for all figures are provided with this paper as a supplementary information file. All image data are available via BioStudies (www.ebi.ac.uk/biostudies) accession number S-BSST1125. Mass data are available via ProteomeXchange (www.ebi.ac.uk/pride) with identifier PXD043101. Molecular docking data are available via BioStudies (www.ebi.ac.uk/biostudies) accession number S-BSST1125.
