# Supplementary material for: PRMT-7/PRMT7 activates HLH-30/TFEB to guard plasma membrane integrity compromised by bacterial pore-forming toxins
Source: Autophagy. 2024 Jan 23;20(6):1335–58. doi: 10.1080/15548627.2024.2306655 (PMC11210913; doi:10.1080/15548627.2024.2306655)
Supplement: PRMT7_supplement_122823 Autophagy R4.docx [file KAUP_A_2306655_SM1952.docx]

Supplementary Materials for

**PRMT-7/PRMT7 activates HLH-30/TFEB to guard plasma membrane integrity compromised by bacterial pore-forming toxins**

Hui-Chen Hsieh *et al.*

*Chang-Shi Chen. Email: cschen@mail.ncku.edu.tw

**This PDF file includes:**

Supplementary text

Figures S1 to S7

Tables S1 to S5

References (1 to 13)

**Other Supplementary Materials for this manuscript include the following:**

Source data to all figures (separate file)

**Supplementary Figures:
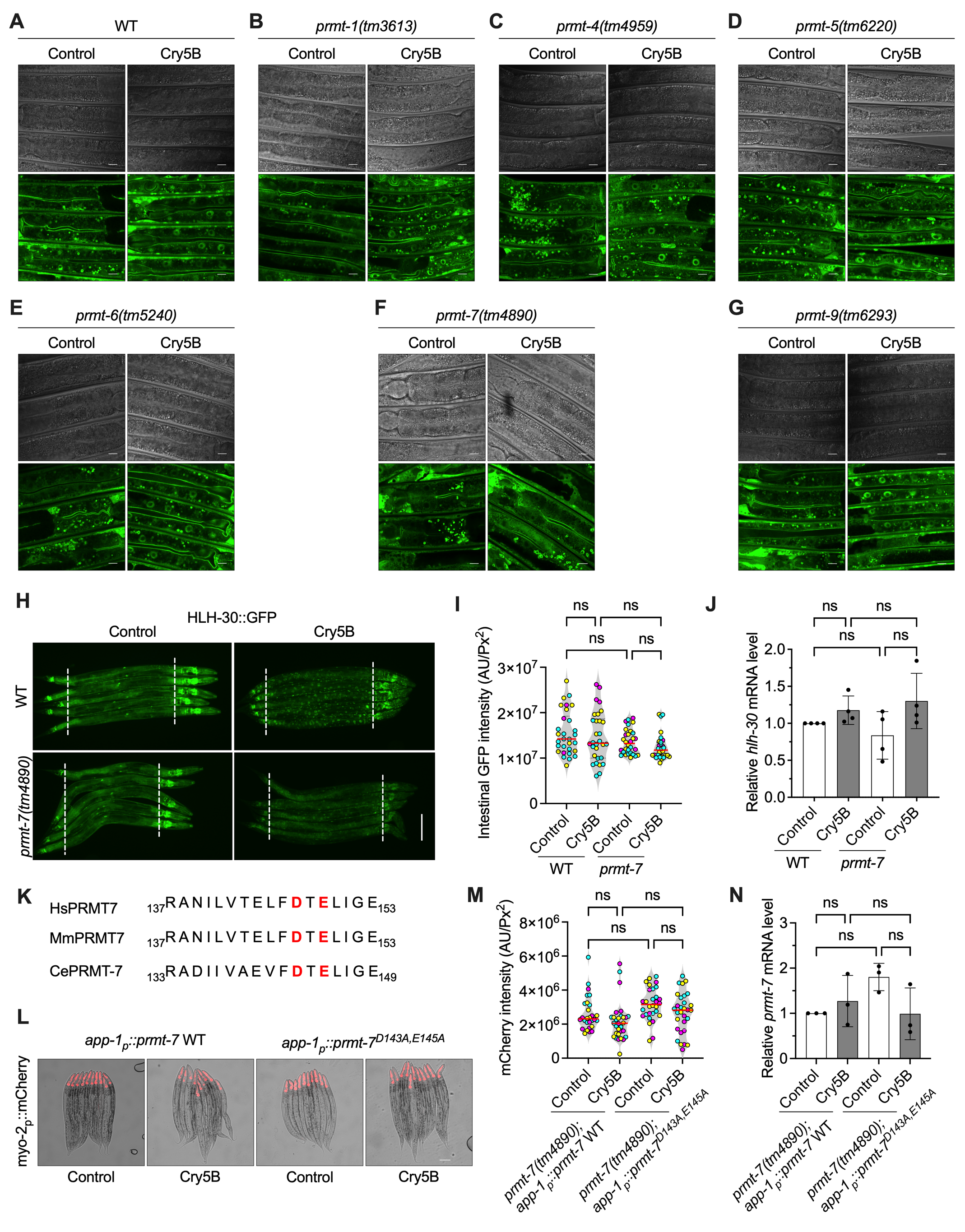
**

**Figure S1.** PRMT7 is the major PRMT paralog that regulates HLH-30 nuclear localization induced by Cry5B-PFT. (**A-G**) The representative DIC (upper) and green fluorescent (lower) images of HLH-30::GFP in the OP433 wild-type (WT), YQ348 *prmt-1(tm3613)*, YQ339 *prmt-4(tm4959)*, YQ352 *prmt-5(tm6220)*, YQ351 *prmt-6(tm5240)*, YQ340 *prmt-7(tm4890)* and YQ353 *prmt-9(tm62933)* mutants exposed to Cry5B or control for 1 h. Scale bars: 10 μm. (**H**) The representative green fluorescent images of HLH-30::GFP in the MAH235 *hlh-30_p_::hlh-30::GFP* and YQ621 *prmt-7(tm4890);hlh-30_p_::hlh-30::GFP* (*prmt-7* mutant) animals exposed to Cry5B or control for 1 h. Intestinal regions are indicated by the white dashed lines. Scale bar: 100 μm. (**I**) The quantification of intestinal GFP intensity in the *hlh-30_p_::hlh-30::GFP* (WT) and *prmt-7(tm4890);hlh-30_p_::hlh-30::GFP* (*prmt-7*) animals exposed to Cry5B or control for 1 h (*n* = 30, *N = 3* in WT-control, *n* = 30, *N = 3* in WT-Cry5B, *n* = 30, *N = 3* in *prmt-7*-control, and *n* = 30, *N = 3* in *prmt-7*-Cry5B). (**J**) The qRT-PCR analysis of *hlh-30* mRNA in N2 wild-type (WT) and FX4890 *prmt-7(tm4890)* mutant animals fed with control (*n* = 12, *N* = 4) or Cry5B (*n* = 12, *N* = 4) for 3 h. (**K**) The amino acid sequences of the catalytic pocket of human PRMT7 (HsPRMT7), mouse PRMT7 (MmPRMT7), and *C. elegans* PRMT-7 (CePRMT-7) were aligned. The essential catalytic aspartate/D and glutamate/E are indicated in red. (**L**) The representative images of *myo-2_p_*::*mCherry* in YQ483 *app-1_p_::prmt-7* WT and YQ482 *app-1_p_::prmt-7^D143A,E145A^* animals exposed to Cry5B or control for 3 h. Scale bars: 100 μm. (**M**) The quantification of mCherry intensity in YQ483 *app-1_p_::prmt-7* WT and YQ482 *app-1_p_::prmt-7^D143A,E145A^* animals exposed to Cry5B or control for 3 h (*n* = 28, *N = 3* in YQ483-control, *n* = 30, *N = 3* in YQ483-Cry5B, *n* = 30, *N = 3* in YQ482-control, and *n* = 30, *N = 3* in YQ482-Cry5B). (**N**) The qRT-PCR analysis of *prmt-7* mRNA in the YQ483 *prmt-7(tm4890);app-1_p_::prmt-7* WT and YQ482 *prmt-7(tm4890);app-1_p_::prmt-7^D143A,E145A^* animals fed with control (each *n* = 9, *N* = 3) or Cry5B (each *n* = 9, *N* = 3) for 3 h. Data information: all data statistics were analyzed by two-way ANOVA. ns represents non-significance. Means are shown in red lines. Each data set of an independent biological repeat is represented by a different color. Source data are available online for this figure.

**
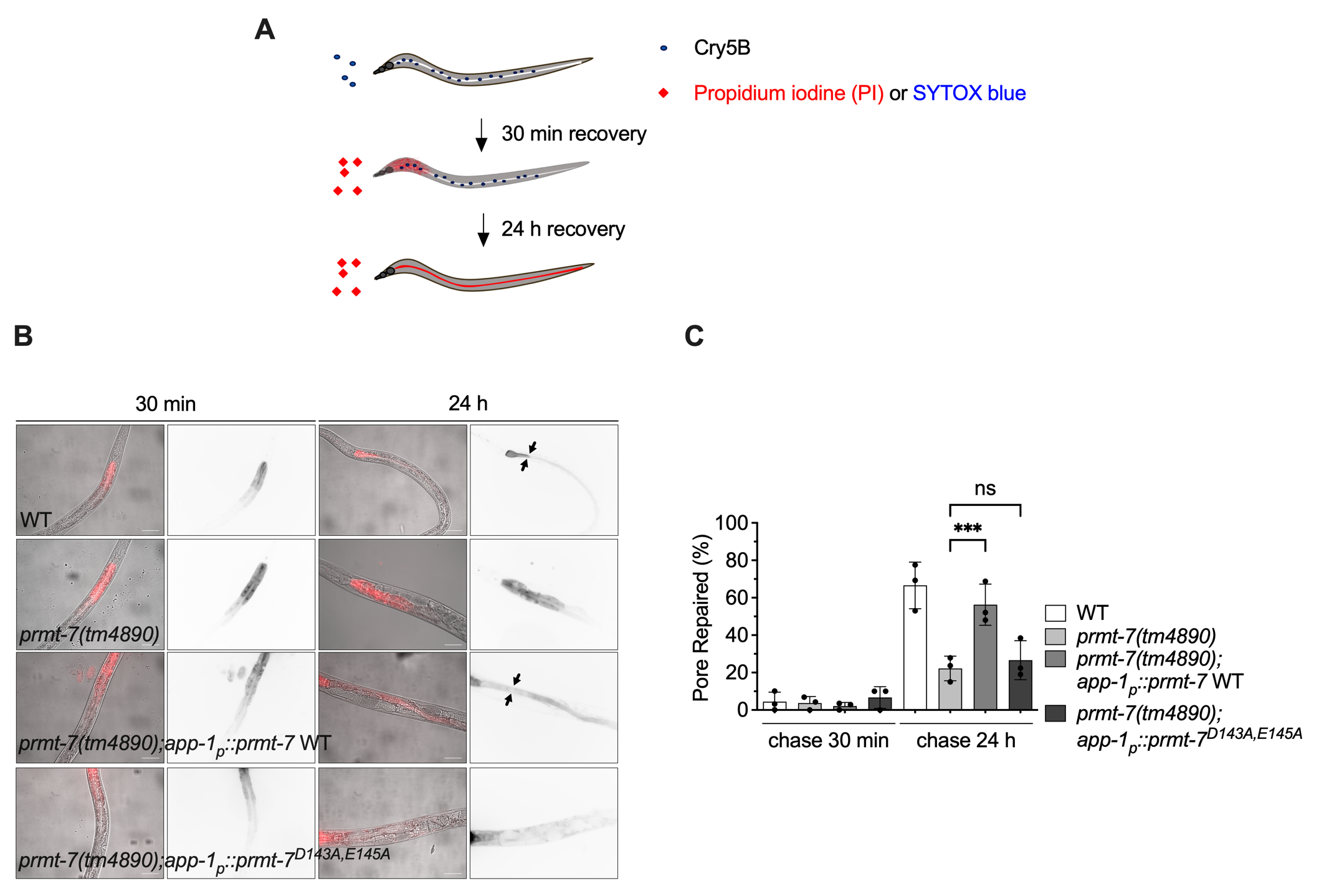
**

**Figure S2.** The pore-repair assay for monitoring the plasma membrane integrity (PMI) of the intestinal cells in *C. elegans*. (**A**) The membrane-impermeable dye propidium iodine (PI) (or SYTOX blue) was used to monitor the PMI and INCED of *C. elegans* intestinal cells in response to PFT intoxication i*n vivo*. After exposing the animals to Cry5B-PFT for 30 min and chasing them briefly on non-Cry5B plates for another 30 min, PI (or SYTOX blue) was able to leak from the intestinal lumen into the cytosol of intestinal cells via the membrane pores generated by Cry5B; thus, these worms showed diffuse PI (or SYTOX blue) signals in the cytosol of intestinal cells. Animals with active INCED, on the contrary, are able to repair the membrane damages caused by Cry5B-PFT after 24 h of recovery on non-Cry5B plates, hence the PI (or SYTOX blue) signals are primarily restricted to the intestinal lumen. (**B**) The representative images of the pore-repair assay. The red signal indicates the distribution of propidium iodine (PI) in the N2 WT, FX4890 *prmt-7(tm4890)*, YQ483 *prmt-7(tm4890);app-1_p_::prmt-7* WT*,* and YQ482 *prmt-7(tm4890);app-1_p_::prmt-7^D143A,E145A^* animals fed with Cry5B for 30 min and transferred to non-Cry5B plates for another 30 min or 24 h for recovery. Scale bars: 50 μm. (**C**) The percentage of pore-repaired animals in the N2 WT (*n* = 90, *N* = 3), YQ340 *prmt-7(tm4890)* (*n* = 90, *N* = 3), YQ483 *prmt-7(tm4890);app-1_p_::prmt-7* WT (*n* = 90, *N* = 3)*,* and YQ482 *prmt-7(tm4890);app-1_p_::prmt-7^D143A,E145A^* (*n* = 90, *N* = 3) after 30 min or 24 h recovery from Cry5B perforation. Data information: all data statistics based on: ****P* <0.001 by two-way ANOVA. Source data are available online for this figure.

**
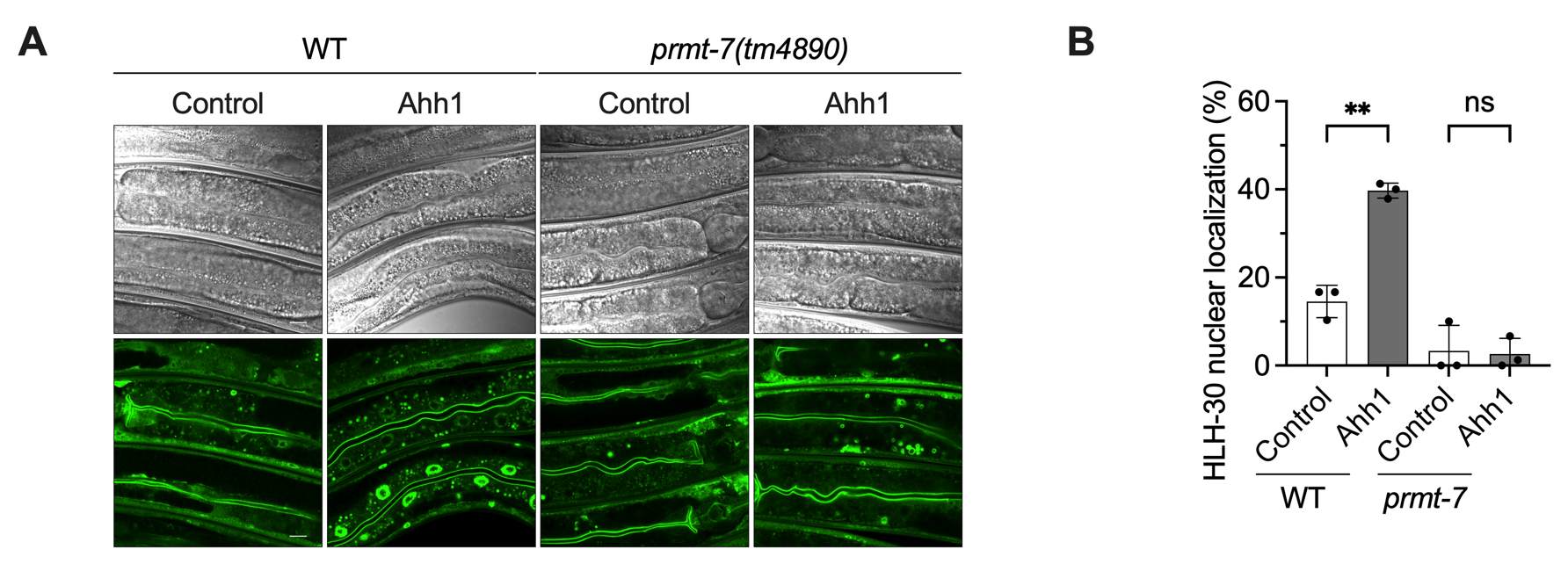
**

**Figure S3.** *prmt-7* is also required for the HLH-30 transnucleation induced by the PFT hemolysin Ahh1 from *Aeromonas dhakensis* in *C. elegans*. (**A**) The representative images of HLH-30::GFP in the wild-type (WT) and YQ340 *prmt-7(tm4890)* animals fed with Ahh1 or control for 3 h. Scale bars: 10 μm. (**B**) The percentage of worms with the HLH-30 nuclear localization signals in the WT and *prmt-7(tm4890)* groups fed with Ahh1 or control for 3 h (*n* = 90, *N =* 3 in WT-control, *n* = 90, *N =* 3 in WT-Ahh1, *n* = 90, *N =* 3 in *prmt-7(tm4890)-*control, and *n* = 90, *N =* 3 in *prmt-7(tm4890)-*Ahh1). Data information: all data statistics based on: ***P* <0.01 by two-way ANOVA. Source data are available online for this figure.

**
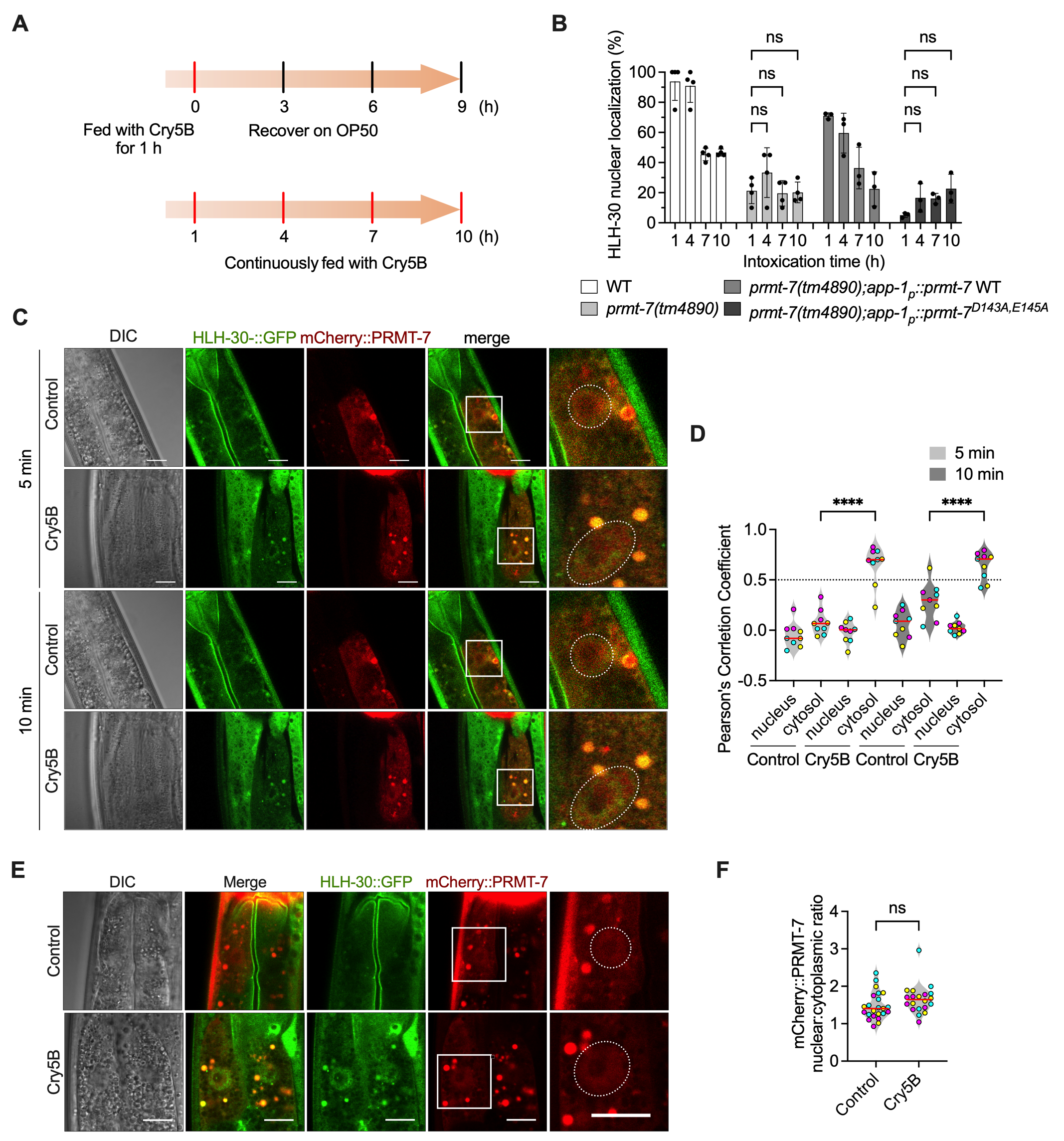
**

**Figure S4.** PRMT-7 is required for the HLH-30 cellular redistribution induced by Cry5B.

(**A**) The diagram of pulse-chase assays. *hlh-30::GFP* worms were either fed with Cry5B for 1 h and then transferred to non-Cry5B plates for 0-, 3-, 6-, and 9-h (upper), or continuously fed with Cry5B for 1-, 4-, 7-, and 10-h (lower). (**B**) The percentage of worms with HLH-30 nuclear localization in OP433 WT (*n* = 120, *N* = 4), YQ340 *prmt-7(tm4890)* (*n* = 120, *N* = 4), YQ483 *prmt-7(tm4890);app-1_p_::prmt-7* WT (*n* = 90, *N* = 3) and YQ482 *prmt-7(tm4890);app-1_p_::prmt-7^D143A,E145A^* (*n* = 90, *N* = 3) animals fed with Cry5B for 1, 4, 7, and 10 h. (**C**) The representative confocal images of HLH-30::GFP and mCherry::PRMT-7 in animals fed with control and Cry5B for 5 and 10 min. Scale bar: 10 μm. (**D**) The Pearson’s correlation coefficient of HLH-30::GFP and mCherry::PRMT-7 in nucleus or cytosol of animals fed with control or Cry5B after 5 and 10 min (*n =* 9 in control-nucleus, *n =* 9 in control-cytosol, *n =* 9 in Cry5B-nucleus, and *n =* 9 in Cry5B-cytosol). Pearson’s correlation coefficient: -1 to 0 indicates negative correlation, 0 to 0.5 indicates no correlation, and 0.5 to 1 indicates positive correlation. (**E**) The representative confocal images of HLH-30::GFP and mCherry::PRMT-7 in YQ454 animals fed with control and Cry5B for 1 h. Scale bar: 10 μm. (**F**) The quantification of the nuclear:cytoplasmic ratio of the mCherry::PRMT-7 intensity in the YQ454 animals fed with Cry5B (*n* = 20) or control (*n* = 23) for 1 h. Data information: all data statistics (except for Fig. S4F) by two-way ANOVA. Statistics in Fig. S4F by unpaired *t* test (two-tailed). **** indicates *P* < 0.0001 by two-way ANOVA. ns represents non-significance. Means are shown in red lines. Each data set of an independent biological repeat was represented by a different color. Source data are available online for this figure**.**


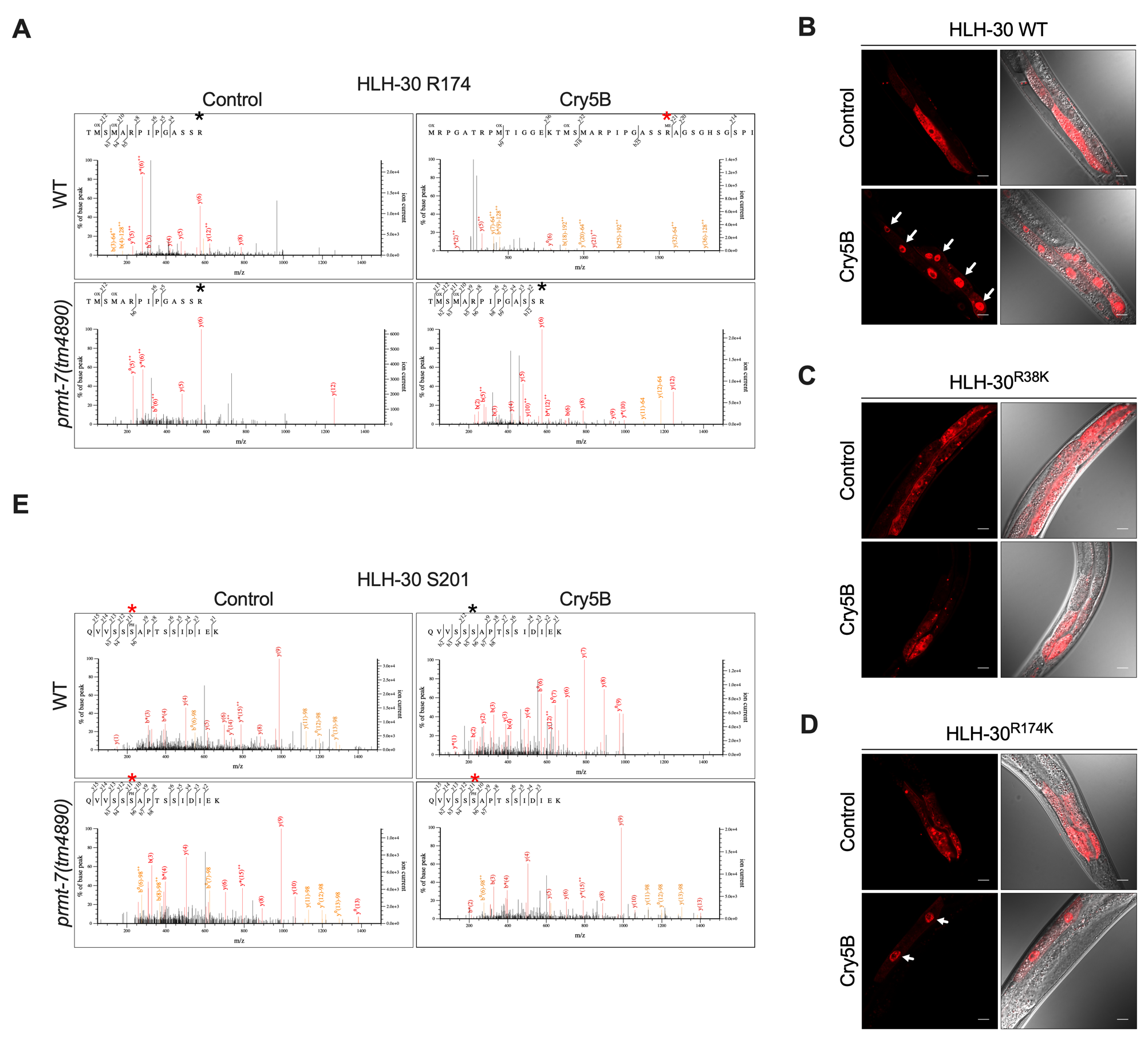


**Figure S5.** HLH-3 R38 methylation is required for the full activation of HLH-30 nuclear localization upon Cry5B intoxication. (**A**) The mass spectrometry results of arginine 174 in HLH-30 from the wild-type (WT) and YQ340 *prmt-7(tm4890)* animals fed with Cry5B or control for 1 h. The black asterisks indicate the non-methylated HLH-30 R174 residues. The red asterisk indicates the methylated HLH-30 R174 residue. (**B-D**) The representative confocal images of HLH-30::mCherry in YQ479 *app-1_p_::hlh-30* WT*::mCherry*, YQ554 *app-1_p_::hlh-30^R38K^::mCherry,* and YQ600 *app-1_p_::hlh-30^R174K^::mCherry* animals fed with Cry5B or control for 1 h. The white arrows indicate nuclear HLH-30::mCherry signals. Scale bar: 10 μm. (E) The mass spectrometry results of serine 201 in HLH-30 from the wild-type (WT) and YQ340 *prmt-7(tm4890)* animals fed with Cry5B or control for 1 h. The black asterisks indicate the non-phosphorylated HLH-30 S201 residue. The red asterisk indicates the phosphorylated HLH-30 S201 residues. Data information: Source data are available online for this figure.

**
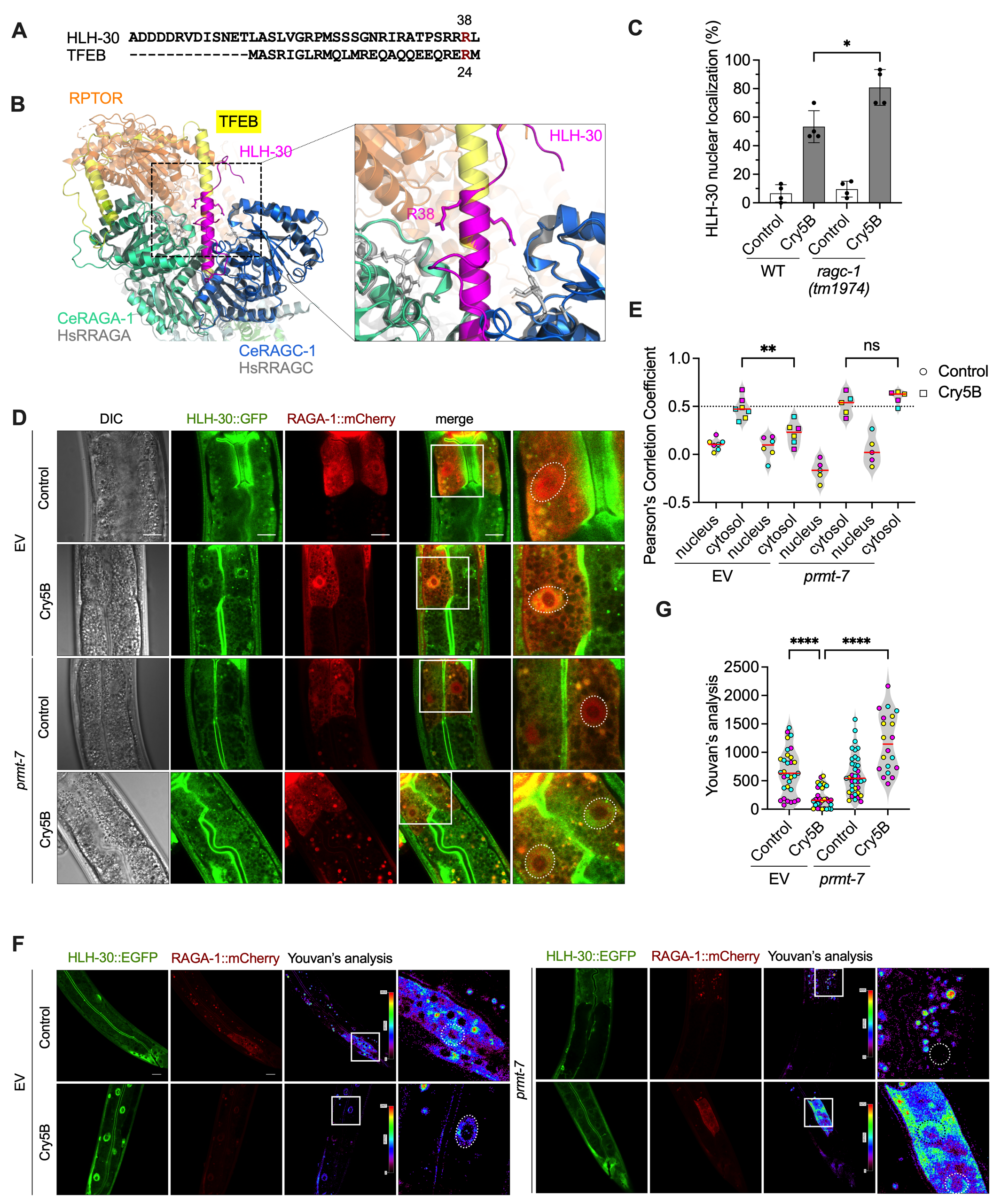
**

**Figure S6.** PRMT-7 is involved in the Cry5B-mediated disruption of HLH-30 and RAGA-1 interactions. (**A**) The amino acid sequence alignment of *C. elegans* HLH-30 and human TFEB. The red Rs represent arginine 38 in HLH-30 and arginine 24 in TFEB, respectively. (**B**) Molecular docking of the *C. elegans* HLH-30, RAGA-1, and RAGC-1 into the resolved TFEB-RRAGA-RRAGC interacting structure [1]. The enlarged figure indicated the potential interaction between arginine 38 of HLH-30 with RAGA-1. (**C**) The percentage of animals with HLH-30::GFP nuclear localization in the WT and YQ592 *ragc-1(tm1974)* worms fed with Cry5B or control for 30 min (*n* = 120, *N* = 4 in WT-control, *n* = 120, *N* = 4 in WT-Cry5B, *n* = 120, *N* = 4 in *ragc-1(tm1974)-*control, and *n* = 120, *N* = 4 in *ragc-1(tm1974)-*Cry5B). (**D**) The representative confocal images of HLH-30::GFP and RAGA-1::mCherry in EV or *prmt-7* RNAi knockdown animals (YQ454) fed with Cry5B or control for 1 h. EV indicates the RNAi empty vector L4440. Enlarged images (right panels) are from the frame of merged HLH-30::GFP and RAGA-1::mCherry images. The dotted circles represent nuclear area. Scale bar: 10 μm. (**E**) The Pearson’s correlation coefficient analysis of RAGA-1::GFP and HLH-30::mCherry signals of EV or *prmt-7* RNAi knockdown animals fed with Cry5B or control for 1 h. EV indicates the RNAi empty vector L4440 (*n* = 7 in EV-control, *n* = 6 in EV-Cry5B, *n* = 5 in *prmt-7-*control, and *n* = 5 in *prmt-7-*Cry5B). Pearson’s correlation coefficient: -1 to 0 indicates negative correlation, 0 to 0.5 indicates no correlation, and >0.5 indicates positive correlation. (**F**) The representative images of the fluorescence resonance energy transfer (FRET) analysis of RAGA-1::EGFP and HLH-30::mCherry signals in EV or *prmt-7* RNAi knockdown animals (YQ454) fed with Cry5B or control for 1 h. EV indicates the RNAi empty vector L4440. The enlarged images (right panels) were from the frame of Youvan’s analysis images. The dotted circles represent nuclear area. Scale bar: 10 μm. (**G**) The quantification of FRET signals between RAGA-1::EGFP and HLH-30::mCherry in the cytosol of intestinal cells by Youvan’s analysis. The EV or *prmt-7* RNAi knockdown animals were exposed to Cry5B or control for 1 h (*n* = 31 in EV-control, *n* = 30 in EV-Cry5B, *n* = 38 in *prmt-7-*control, and *n* = 20 in *prmt-7-*Cry5B). EV indicates the RNAi empty vector L4440. Data information: all data statistics based on: **P* <0.05, ***P* < 0.01 and *****P* < 0.0001 by two-way ANOVA. Means are shown in red lines. Each data set of an independent biological repeat was represented by a different color. Source data are available online for this figure.

**
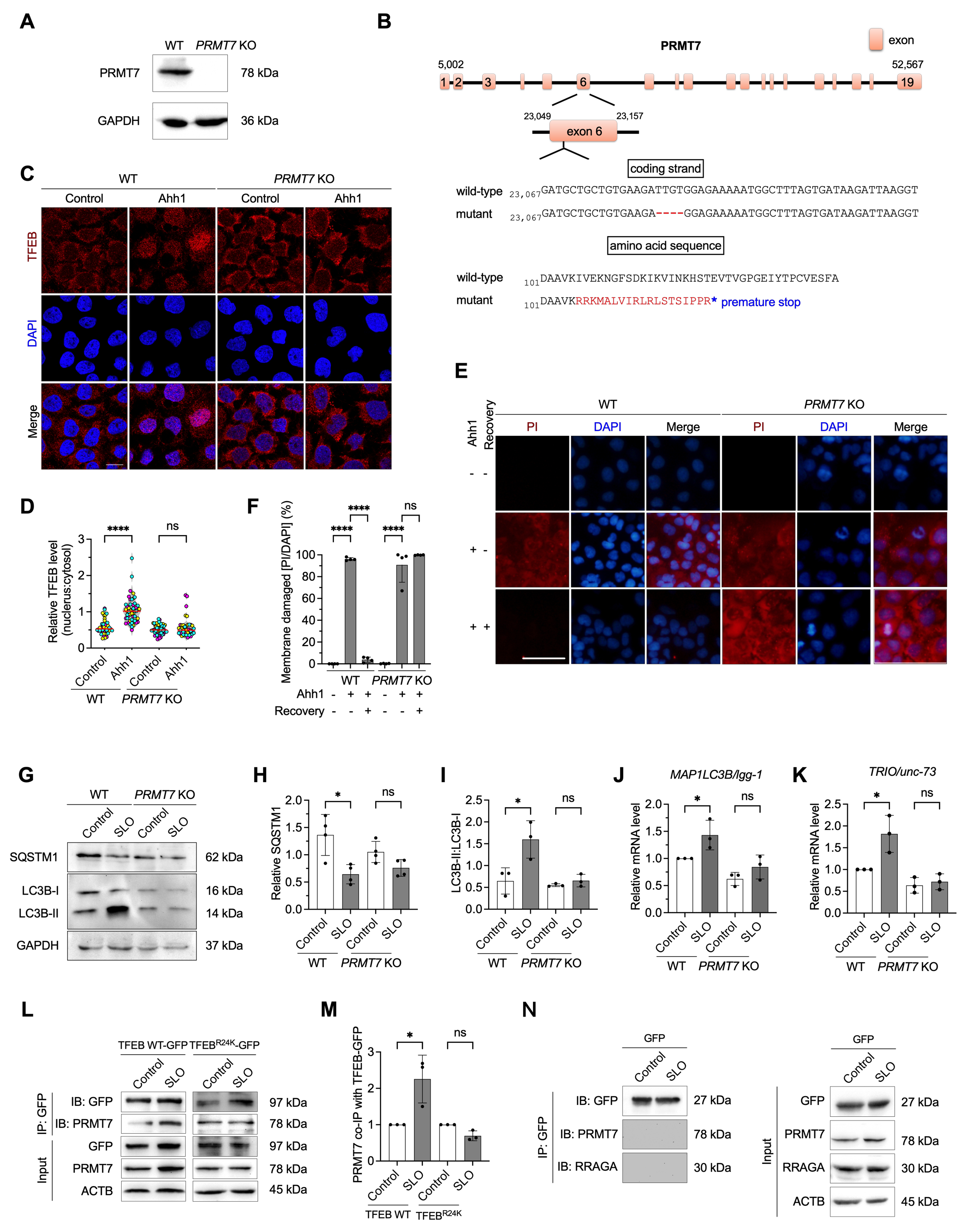
**

**Figure S7.** PRMT7 is also required for the TFEB nuclear localization and the INCED activation elicited by PFT in human cells. (**A**) The representative WB images of PRMT7 and GAPDH in WT and *PRMT7* KO cells. (**B**) The diagram of Sanger sequencing results of the Caco-2 *PRMT7* KO cell line. Red dashes indicate a 4 nucleotides deletion in the *PRMT7* KO cell. Red amino acid abbreviations indicate frame shifting resulting from the nucleotides deletion. Blue asterisk represents a premature stop. (**C**) The representative confocal images of TFEB in WT and *PRMT7* KO cells treated with Ahh1 or control for 9 h. DAPI indicates nucleus staining. Scale bar: 20 μm. (**D**) The quantification of the nuclear:cytoplasmic ratio of the TFEB intensity in WT and *PRMT7* KO cells treated with Ahh1 or control for 9 h (*n* = 43 in WT-control, *n* = 50 in WT-Ahh1, *n* = 50 in *PRMT7* KO-control, *n* = 43 in *PRMT7* KO-Ahh1). (**E**) The representative images of the pore-repair assay. The red signal indicates the distribution of propidium iodine (PI) in the WT and *PRMT7* KO cells treated with Ahh1 or control for 9 h. DAPI indicates nucleus staining. Scale bar: 20 μm. (**F**) The percentage of membrane damaged cells in WT and *PRMT7* KO cell infected with Ahh1 or control for 9 h (*n* =1007, *N* = 4 in WT-control, *n* = 715, *N* = 4 in WT-Ahh1, *n* = 1002 , *N* = 4 in WT-Ahh1 with recovery, *n* = 1211, *N* = 4 in *PRMT7* KO-control, *n* = 1035, *N* = 4 in *PRMT7* KO-Ahh1, *n* = 994, *N* = 4 in *PRMT7* KO-Ahh1 with recovery). (**G**) The representative WB images of SQSTM1, LC3B and GAPDH from WT and *PRMT7* KO cells treated with SLO or control for 75 min. (H) The quantification of SQSTM1 expression in WT and *PRMT7* KO cells treated with SLO or control for 75 min (*N* = 3). The fold change of SQSTM1 is normalized with GAPDH. (**I**) The quantification of LC3B expression in WT and *PRMT7* KO cells treated with SLO or control for 75 min (*N* = 3). (**J-K**) The qRT-PCR analysis of *MAP1LC3B/lgg-1* (*n* = 9*, N* = 3) and *TRIO*/*unc-73* (*n* = 9*, N* = 3) mRNA in the WT and *PRMT7* KO cells treated with SLO or control for 50 min. (**L**) The representative WB images of co-immunoprecipitation of TFEB-GFP and PRMT7 in TFEB WT and TFEB^R24K^ cell. (**M**) The quantification of PRMT7 pulled-down with TFEB-GFP in TFEB WT and TFEB^R24K^ cell (*N* = 3). The fold change of co-IP PRMT7 is normalized with immunoprecipitated TFEB-GFP. (**N**) The representative WB images of co-immunoprecipitation of GFP with either PRMT7 or RRAGA in HeLa cells expressing GFP. Data information: all data statistics except Fig. S7M based on: **P* <0.05 and *****P* < 0.0001 by two-way ANOVA. Fig. S7M statistic based on: **P* <0.05 by unpaired *t* test (two-tailed). ns represents non-significance. Means are shown in red lines. Each data set of an independent biological repeat was represented by a different color. Source data are available online for this figure.

**Supplementary Tables:**

**Table S1.** *C. elegans* strains.

| Description/Genotype | Sources | Identifier |
| --- | --- | --- |
| wild type N2 | Caenorhabditis Genetics Center (CGC) [2] | Strain: Bristol N2 |
| *unc-119(tm4063);wgIs433[hlh-30::TY1::EGFP::3xFLAG+unc-119(+)]* | Caenorhabditis Genetics Center (CGC) [3] | Strain: OP433 |
| *sqIs19 [hlh-30p::hlh-30::GFP + rol-6(su1006)]* | Caenorhabditis Genetics Center (CGC) [4] | Strain: MAH235 |
| *adls2122[lgg-1_p_::lgg-1*-GFP *+ rol-6(su1006)]* | Caenorhabditis Genetics Center (CGC) [5] | Strain: DA2123 |
| *rpl-43(bp399);bpIs151[SQST-1::GFP]* | Caenorhabditis Genetics Center (CGC) [6] | Strain: HZ946 |
| *raga-1(ok386)* | Caenorhabditis Genetics Center (CGC) | Strain: VC222 |
| *ragc-1(tm1974)* | National Bio-Resource Project (NBRP) [7] | Strain: FX1974 |
| *hlh-30(tm1978)* | National Bio-Resource Project (NBRP) | Strain: FX1978 |
| *unc-119(tm4063)* | National Bio-Resource Project (NBRP) | Strain: FX4063 |
| *prmt-1(tm3613)* | National Bio-Resource Project (NBRP) | Strain: FX3613 |
| *prmt-4(tm4595)* | National Bio-Resource Project (NBRP) | Strain: FX4595 |
| *prmt-5(tm6620)* | National Bio-Resource Project (NBRP) | Strain: FX6620 |
| *prmt-6(tm5240)* | National Bio-Resource Project (NBRP) | Strain: FX5240 |
| *prmt-7(tm4890)* | National Bio-Resource Project (NBRP) | Strain: FX4890 |
| *prmt-9(tm6293)* | National Bio-Resource Project (NBRP) | Strain: FX6293 |
| *prmt-1(tm3613);wgls433[hlh-30:TY1::EGFP::3xFLAG+unc-119(+)]* | This study | Strain: YQ348 |
| *prmt-4(tm4959);wgls433[hlh-30:TY1::EGFP::3xFLAG+unc-119(+)]* | This study | Strain: YQ339 |
| *prmt-5(tm6220);wgls433[hlh-30:TY1::EGFP::3xFLAG+unc-119(+)]* | This study | Strain: YQ352 |
| *prmt-6(tm5240);wgls433[hlh-30:TY1::EGFP::3xFLAG+unc-119(+)]* | This study | Strain: YQ351 |
| *prmt-7(trm4890);wgls433[hlh-30:TY1::EGFP::3xFLAG+unc-119(+)]* | This study | Strain: YQ340 |
| *prmt-9(tm6293);wgls433[hlh-30:TY1::EGFP::3xFLAG+unc-119(+)]* | This study | Strain: YQ353 |
| *unc-119(tm4063);wfEx351[prmt-7_p_::dGFP::unc-54+unc119(+)]* | This study | Strain: YQ432 |
| *prmt-7(tm4890);unc-119(tm4063);wgls433[hlh-30::TY1::EGFP::3xFLAG+unc-119(+)];wfEx409[app-1_p_::prmt-7^D143A,E145A^::unc-54];myo-2_p_::mCherry::myo-2* | This study | Strain: YQ482 |
| *prmt-7(tm4890);unc-119(tm4063);wgls433[hlh-30::TY1::EGFP::3xFLAG+unc-119(+)];wfEx503[app-1_p_::prmt-7 WT::unc-54];myo-2_p_::mCherry::myo-2* | This study | Strain: YQ483 |
| *prmt-7(tm4890);sqIs19 [hlh-30p::hlh-30::GFP+rol-6(su1006)]* | This study | Strain: YQ621 |
| *prmt-7(tm4890);adls2122[lgg-1_p_::lgg-1*-GFP*+rol-6(su1006)]* | This study | Strain: YQ349 |
| *unc-119(tm4063);wgls433[hlh-30::TY1::EGFP::3xFLAG+unc-119(+)];wfEx442[app-1_p_::mCherry::prmt-7];myo-2_p_::mCherry::myo-2* | This study | Strain: YQ454 |
| *hlh-30(tm1978);unc-119(tm4063);wfEx407[app-1_p_::hlh-30^WT^::mCherry::unc-54+unc-119(+)];myo-2_p_::GFP::myo-2* | This study | Strain: YQ479 |
| *hlh-30(tm1978);unc-119(tm4063);wfEx467[app-1_p_::hlh-30^R38K^::mCherry::unc-54;unc-119(+)];myo-2_p_::GFP::myo-2* | This study | Strain: YQ554 |
| *hlh-30(tm1978);unc-119(tm4063);wfEx504[app-1_p_::hlh-30^R174K^::mCherry::unc-54;unc-119(+)];myo-2_p_::GFP::myo-2* | This study | Strain: YQ600 |
| *raga-1(ok386);unc-119(tm4063);wgls433[hlh-30::TY1::EGFP::3xFLAG+unc-119(+)]* | This study | Strain: YQ534 |
| *ragc-1(tm1974); unc-119(tm4063);wgls433[hlh-30::TY1::EGFP::3xFLAG+unc-119(+)]* | This study | Strain: YQ592 |
| *unc-119(tm4063);wgls433[hlh-30::TY1::EGFP::3xFLAG+unc-119(+)];wfEx473[app1_p_::raga-1::mCherry::raga-1];myo-2_p_::mCherry::myo-2* | This study | Strain: YQ565 |
| *hlh-30(tm1978);unc-119(tm4063);wfEx467[app-1_p_::hlh-30^R38K^::mCherry::unc-54+unc-119(+)];myo-2_p_::GFP::myo-2;wfEx493[vha-6_p_::raga-1::EGFP::raga-1+rol-6(su1006)]* | This study | Strain: YQ596 |
| *hlh-30(tm1978);unc-119(tm4063);wfEx407[app-1_p_::hlh-30^WT^::mCherry::unc-54+unc-119(+)];myo-2_p_::GFP::myo-2;wfEx493[vha-6_p_::raga-1::EGFP::raga-1+rol-6(su1006)]* | This study | Strain: YQ601 |

**Table S2.** Bacteria strains.

| Description | Sources | Identifier |
| --- | --- | --- |
| *E. coli*. uracil auxotrophy, standard *C. elegans* laboratory food source. | Caenorhabditis Genetics Center (CGC) [2] | Strain: OP50 |
| *E. coli* OP50 carrying pQE9 | This study | Strain: YQ602 |
| *E. coli* OP50 carrying pQE9-Cry5B | This study | Strain: YQ603 |
| *E. coli* OP50 carrying pQE9-Cry21A | This study | Strain: YQ604 |
| *E. coli* XL-10-Gold carrying pQE9 | This study | Strain: YQ605 |
| *E. coli* XL-10-Gold carrying pQE9-Cry5B | This study | Strain: YQ606 |
| *E. coli* XL-10-Gold carrying pQE9-Cry21A | This study | Strain: YQ607 |
| *E. coli* BL21 | Bioresource Collection and Research Center (BCRC) | Strain: BL21 |
| *E. coli* BL21 carrying pET30a-Ahh1 | This study | Strain: PL101 |
| *E. coli* HT115 carrying L4440, an empty vector, used as negative control of RNAi | Caenorhabditis Genetics Center (CGC) [8] | L4440 |
| *E. coli* HT115 expressing *prmt-7* RNAi | Ahringer RNAi library | WS225 I-4B23 |
| *E. coli* HT115 expressing *xpo-1* RNAi | Ahringer RNAi library | WS225 V-5L21 |
| *E. coli* HT115 expressing *ima-2* RNAi | Ahringer RNAi library | WS225 I-3C11 |
| *E. coli* HT115 expressing *let-363* RNAi | Ahringer RNAi library | Supplement 3_B12 |
| *E. coli* HT115 expressing *ftt-2* RNAi | Ahringer RNAi library | WS225 X-5F07 |

**Table S3.** Plasmids.

| Description | Sources | Identifier |
| --- | --- | --- |
| Plasmid: an empty vector was used as negative control of RNAi | Caenorhabditis Genetics Center (CGC) | L4440 |
| Plasmid: *prmt-7_p_::dGFP::unc-54+unc119(+)* | This study | pwf351 |
| Plasmid: *app-1_p_::prmt-7^D143A,E145A^::unc-54* | This study | pwf409 |
| Plasmid: *app-1_p_::prmt-7 WT::unc-54* | This study | pwf503 |
| Plasmid: *app-1_p_::mCherry::prmt-7* | This study | pwf442 |
| Plasmid: *app-1_p_::hlh-30 WT::mCherry::unc-54+unc-119(+)* | This study | pwf407 |
| Plasmid: *app-1_p_::hlh-30^R38K^::mCherry::unc-54+unc-119(+)* | This study | pwf447 |
| Plasmid: *app-1_p_::hlh-30^R174K^::mCherry::unc-54+unc-119(+)* | This study | pwf504 |
| Plasmid: *app1_p_::raga-1::mCherry::raga-1* | This study | pwf473 |
| Plasmid: *vha-6_p_::raga-1::EGFP::raga-1* | This study | pwf493 |
| Plasmid: DD-Cas9 with filler sequence and Venus (EDCPV) | Addgene [9] | 90085 |
| Plasmid: pEGFP-N1-TFEB | Addgene [10] | 38119 |
| Plasmid: pEGFP-N1-TFEB^R8K^ | This study | pwf512 |
| Plasmid: pEGFP-N1-TFEB^R24K^ | This study | pwf513 |

**Table S4.** Primers.

| Description | Sequences | Sequences |
| --- | --- | --- |
| *prmt-7* qRT PCR | Forward: GCCAGAAGTCGATTTGGAGA | Reverse: CCTTCTCGAGCAGCCATTAG |
| *lgg-1* qRT PCR | Forward: GAAGAAGTACTTGGTCCCATCCG | Reverse: CGTGATGGTCCTGGTAGAGTTG |
| *atg-18* qRT PCR | Forward: CTTACACATCTTGGTCTCAAC | Reverse: CTTACACATCTTGGTCTCAAC |
| *ced-1* qRT PCR | Forward: GGTGAAGACGATTGTGGACGAC | Reverse: TTGTCCGCGTTTCTCAACAG |
| *unc-73* qRT PCR | Forward: AAATCGTTGCGACGAATCTTC | Reverse: CGATTCAGTAGTATGTCCGTTGGC |
| *eft-2* qRT PCR | Forward: TCGAAATTCAATGCCCAGAA | Reverse: CTCCTCGAAAACGTGTCCTCTT |
| *gapdh* qRT PCR | Forward: ACCAGCCTCAAGATCATCAGCA | Reverse: TGCTAAGCAGTTGGTGGTGC |
| *MAP1LC3B/lgg-1* qRT PCR | Forward: CGCACCTTCGAACAAAGAGTAG | Reverse:  TGACATGGTCAGGTACAAGG |
| *TRIO*/*unc-73* qRT PCR | Forward: GCTTATTCTGAGGTCAGCCAAG | Reverse: GTAGTTGGCAGAGGCTGTC |
| PRMT-7^D143A,E145A^ site-directed mutagenesis | Forward: TATTTGCGACAGCGTTAATTGGAGAAGGAGCTC | Reverse: ACGCTGTCGCAAATACCTCTGCTACAATAGTATC |
| HLH-30^R38K^ site-directed mutagenesis | Forward: CACCCCATCCAGACGGAACTGGTT TCTGCGCCG | Reverse: CGGCGCAGAAACCAGTTTCCGTCTGGATGGGGTG |
| HLH-30^R174K^ site-directed mutagenesis | Forward: CAGGCGCCAGCTCCAAGGCTGGCTCAGGAC | Reverse: GTCCTGAGCCAGCCTTGGAGCTGGCGCCTG |
| EGFP-N1-TFEB^R8K^ site-directed mutagenesis | Forward: CACGCATAGGGTTGAAGATGCAGCTCATGCGG | Reverse: CCGCATGAGCTGCATCTTCAACCCTATGCGTG |
| EGFP-N1-TFEB^R24K^ site-directed mutagenesis | Forward: ACGGGAGAAGATGCAGCAAC | Reverse: TGCATCTTCTCCCGTTGCTC |

**Supplementary References:**

[1] Cui Z, Napolitano G, de Araujo MEG, et al. Structure of the lysosomal mTORC1-TFEB-Rag-Ragulator megacomplex. Nature. 2023 Feb;614(7948):572-579.

[2] Brenner S. The genetics of Caenorhabditis elegans. Genetics. 1974 May;77(1):71-94.

[3] Sarov M, Schneider S, Pozniakovski A, et al. A recombineering pipeline for functional genomics applied to Caenorhabditis elegans. Nat Methods. 2006 Oct;3(10):839-44.

[4] Lapierre LR, De Magalhaes Filho CD, McQuary PR, et al. The TFEB orthologue HLH-30 regulates autophagy and modulates longevity in Caenorhabditis elegans. Nat Commun. 2013;4:2267.

[5] Kang C, You YJ, Avery L. Dual roles of autophagy in the survival of Caenorhabditis elegans during starvation. Genes Dev. 2007 Sep 1;21(17):2161-71.

[6] Guo B, Huang X, Zhang P, et al. Genome-wide screen identifies signaling pathways that regulate autophagy during Caenorhabditis elegans development. EMBO Rep. 2014 Jun;15(6):705-13.

[7] Mitani S. Nematode, an experimental animal in the national BioResource project. Exp Anim. 2009 Jul;58(4):351-6.

[8] Timmons L, Fire A. Specific interference by ingested dsRNA. Nature. 1998 Oct 29;395(6705):854.

[9] Senturk S, Shirole NH, Nowak DG, et al. Rapid and tunable method to temporally control gene editing based on conditional Cas9 stabilization. Nat Commun. 2017 Feb 22;8:14370.

[10] Roczniak-Ferguson A, Petit CS, Froehlich F, et al. The transcription factor TFEB links mTORC1 signaling to transcriptional control of lysosome homeostasis. Sci Signal. 2012 Jun 12;5(228):ra42.

**Source Data (separate file)**

Source data are available online.
